# 5-Ethynyl-2′-deoxycytidine and 5-ethynyl-2′-deoxyuridine are differentially incorporated in cells infected with HSV-1, HCMV, and KSHV viruses

**DOI:** 10.1074/jbc.RA119.012378

**Published:** 2020-03-23

**Authors:** Salomé Manska, Rionna Octaviano, Cyprian C. Rossetto

**Affiliations:** Department of Microbiology and Immunology, University of Nevada, Reno, School of Medicine, Reno, Nevada 89557

**Keywords:** nucleoside/nucleotide analogue, nucleoside/nucleotide metabolism, herpesvirus, DNA replication, viral replication, 5-ethynyl-2′-deoxycytidine (EdC), 5-ethynyl-2′-deoxyuridine (EdU), thymidine kinase, pulse labeling, cell proliferation, non-radioactive labeling, thymidine kinase, fluorescent reporter

## Abstract

Nucleoside analogues are a valuable experimental tool. Incorporation of these molecules into newly synthesized DNA (*i.e.* pulse-labeling) is used to monitor cell proliferation or to isolate nascent DNA. Some of the most common nucleoside analogues used for pulse-labeling of DNA in cells are the deoxypyrimidine analogues 5-ethynyl-2′-deoxyuridine (EdU) and 5-ethynyl-2′-deoxycytidine (EdC). Click chemistry enables conjugation of an azide molecule tagged with a fluorescent dye or biotin to the alkyne of the analog, which can then be used to detect incorporation of EdU and EdC into DNA. The use of EdC is often recommended because of the potential cytotoxicity associated with EdU during longer incubations. Here, by comparing the relative incorporation efficiencies of EdU and EdC during short 30-min pulses, we demonstrate significantly lower incorporation of EdC than of EdU in noninfected human fibroblast cells or in cells infected with either human cytomegalovirus or Kaposi's sarcoma-associated herpesvirus. Interestingly, cells infected with herpes simplex virus type-1 (HSV-1) incorporated EdC and EdU at similar levels during short pulses. Of note, exogenous expression of HSV-1 thymidine kinase increased the incorporation efficiency of EdC. These results highlight the limitations when using substituted pyrimidine analogues in pulse-labeling and suggest that EdU is the preferable nucleoside analogue for short pulse-labeling experiments, resulting in increased recovery and sensitivity for downstream applications. This is an important discovery that may help to better characterize the biochemical properties of different nucleoside analogues with a given kinase, ultimately leading to significant differences in labeling efficiency of nascent DNA.

## Introduction

**Figure 1.**
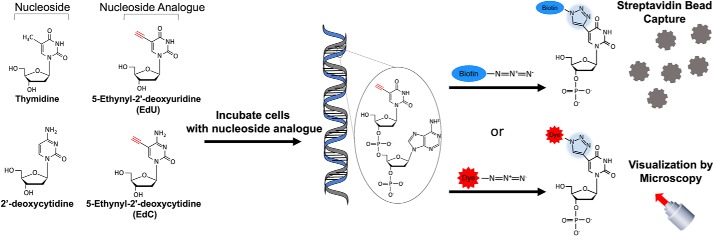
**Method for isolating and labeling nascent DNA using nucleoside analogues EdU and EdC.** Structure comparison of thymidine and 2′-deoxycytidine with corresponding nucleoside analogues EdU and EdC. The nucleoside analogues carry an ethynyl group (alkyne) in *red*. Incubation with the nucleoside analogue during DNA replication leads to incorporation of EdU or EdC into nascent DNA. An azide molecule tagged with biotin or a fluorescent dye can be conjugated to the nucleoside analogue by copper-catalyzed azide-alkyne cycloaddition (click chemistry) (highlighted in *blue*). Labeled nascent DNA is tagged with biotin for streptavidin bead capture or a fluorescent dye for microscopy.

Nucleoside analogues have a wide variety of applications in experimental biology, ranging from monitoring rates of cell proliferation to isolating replication forks (1, 2). They retain the basic nucleoside structure, which allows for incorporation into newly synthesized DNA, and have additional modifications or chemical additions that make newly synthesized DNA (or proliferating cells) distinguishable from the nonreplicating DNA (or quiescent cells). The early techniques for assessing cell proliferation used radioactive tritium-labeled thymidine (3). These analogues were eventually replaced with nonradioactive analogues, such as 5-bromo-2′-deoxyuridine (BrdU),^2^ which are identified using a specific mAb (3, 4). One of the experimental considerations and drawbacks when using BrdU is the size of the antibody that is ultimately required to recognize the nucleoside analogue. In order for the BrdU epitope to be accessible to the antibody, harsh denaturing conditions are applied. These conditions result in the loss of native tissue and protein conformations, making additional protein immunofluorescence analysis difficult. Alternatives to radiolabeled thymidine and BrdU include the more recently developed nucleoside analogues EdU and EdC. These analogues contain a terminal alkyne group in the 5-position that can be conjugated to azide-containing molecules via copper-catalyzed azide-alkyne “click” chemistry (Fig. 1) (5–7). The addition of the azide molecule to the terminal alkyne does not require harsh denaturing conditions as required for the detection of BrdU, thereby allowing identification and isolation of nascent DNA along with associated proteins. The drawback to using EdU and, to a lesser extent EdC, is the potential for cytotoxicity resulting in a decrease in cell proliferation. The cytotoxicity associated with EdU comes from the uracil base, as it is not typically found in DNA. Incorporation of a uracil base results in recognition of the rogue nucleotide and repair by host machinery.

At the most basic level, the ability to accurately replicate DNA depends on the integrity of the template, fidelity of the synthesis enzymes, and availability of correct nucleotides. For DNA synthesis, the nucleosides used are deoxyadenosine (A), deoxyguanosine (G), deoxycytidine (C), and deoxythymidine (T). Thymidine is formed through the addition of a methyl group to the uracil base by thymidylate synthase, turning deoxyuridine monophosphate (dUMP) to deoxythymidine monophosphate (dTMP). Uracil is seldom found in DNA but can occur during DNA synthesis through incorrect incorporation of deoxyuridine in place of thymidine or through a deamination reaction of cytosine. The deamination of cytosine (which base-pairs with guanine) to uracil (which base-pairs with adenine) would cause a G:C to A:T transition, resulting in a DNA point mutation. There are multiple cellular mechanisms to prevent and repair uracil in the DNA. The main mechanisms are deoxyuridine triphosphatase (dUTPase), which reduces the available pools of dUTP by converting dUTP to dUMP, and uracil-DNA glycosylase (UNG), which removes incorporated uracil through the base excision repair pathway (8–11).

Many of the nucleoside analogues that are routinely used in experimental cell labeling assays were originally developed as antiviral or chemotherapeutic agents (12–15). As EdU had potential therapeutic interest, initial experiments documented cytotoxicity associated with long-term exposure of EdU (14, 16–20). It is thought that the cytotoxicity results from two main causes. One cause is the inhibition of thymidylate synthetase, a pivotal DNA biosynthesis enzyme necessary to catalyze the conversion of dUMP to dTMP and convert 5,10-methylenetetrahydrofolate to 7,8-dihydrofolate (13, 21). The second is through incorporation of the EdU into DNA, which results in the induction of the DNA damage signaling pathway, where UNG causes a single-stranded break during base excision of the uracil (22, 23). Triggering this pathway inhibits cell cycle progression and can ultimately lead to apoptosis if unresolved (16, 17, 19, 20). To reduce the cytotoxicity associated with EdU, while still retaining the ability to label DNA, many prefer to use EdC (5). Many assume that EdC can be used in place of EdU, but EdC is not without its own drawbacks and considerations. One study found that EdC is deaminated to form EdU before being incorporated into DNA and presents toxicity corresponding to the formation of EdU (24).

In recent years, one of the more innovative uses of EdU has been to isolate newly synthesized DNA along with associated proteins using a technique called isolation of proteins on nascent DNA (iPOND) and accelerated native iPOND (aniPOND) (1, 2, 25). Using specific pulse or pulse-chase time course protocols, this powerful technique has provided new insight into the spatiotemporal dynamics of proteins associating with and dissociating from the active replication forks during DNA synthesis. iPOND and aniPOND have also been used for studying replication of DNA viruses and to identify proteins participating in viral DNA replication (26–30). Because all viruses are obligate intracellular parasites, which rely on the host to varying degrees, it is essential to determine the optimal nucleoside analogue labeling conditions that result in the highest yield of labeled viral DNA while minimizing the amount of cellular DNA. If the enrichment of cellular DNA is not minimized, it is very difficult to interpret the results of the cellular proteins in the aniPOND experiments, as cellular proteins could be present on either cellular or viral DNA. A previous report using iPOND to identify cellular proteins during replication of adenovirus, herpes simplex virus type-1 (HSV-1), and vaccinia virus performed normalization strategies between infected and mock-infected cells to identify cellular proteins enriched at viral replication forks (29). This study used next-generation sequencing to calculate the percentage of cellular reads from the total isolated EdU-labeled DNA. It reported that during a 15-min EdU pulse in infected U2OS cells, the percentage aligned to the human genome was 28.5% for adenovirus, 45.6% for HSV-1, and 43.5% for vaccinia virus. Sequences that did not align to the human genome were inferred to be viral DNA (29). The percentages that align to the human genome demonstrate a significant amount of captured cellular DNA and suggest that there can be additional optimization of the protocols to reduce those amounts and increase the viral DNA/cellular DNA enrichment ratio.

To apply the aniPOND technique to study proteins associated with viral DNA synthesis, our initial objective was to optimize the nascent DNA labeling conditions to increase the enrichment of viral DNA and reduce the amount of cellular DNA. We tested several experimental parameters that would contribute to the preferential enrichment of viral DNA. Because HCMV (along with many herpesviruses) contains a genome that is GC-rich, we assumed that the incorporation and enrichment of viral DNA would be greater using EdC compared with EdU. We focused on short time pulses (30 min) to capture active replication forks, whereas longer pulses would result in increased amounts of labeled mature DNA. We were surprised to find that during the short 30-min pulses, there was significantly lower incorporation of EdC compared with EdU in noninfected cells or cells infected with HCMV or KSHV. Interestingly, cells infected with HSV-1 showed similar levels of incorporation for EdC and EdU. Additional investigation and experiments identified HSV-1 thymidine kinase (TK) as the factor that was allowing for increased incorporation efficiency of EdC in HSV-1–infected cells. Furthermore, we demonstrated that the deficiency in EdC incorporation is partly overcome by exogenous expression of HSV-1 TK. Using an *in vitro* kinase assay, we show that there are significant differences in the phosphorylation rates between EdU and EdC that contribute to lower EdC enrichment. In short pulse-labeling experiments, there was no associated cytotoxicity with EdU or EdC. Efficient incorporation of EdC during short time pulse was only observed in cells that were infected with HSV-1 or expressing HSV-TK. Overall, we determined that EdU is the preferable nucleoside analogue for short pulse-labeling experiments, resulting in increased DNA labeling and sensitivity for downstream applications.

## Results

### Efficient incorporation of EdU but not EdC after 30-min pulse-labeling in normal human fibroblasts

One of the considerations for any experiment focused on viral replication is to use an appropriate cell culture model that contains cells that are both susceptible and permissive to infection. Normal human fibroblasts (HFs) support lytic replication of both HCMV and HSV-1. The advantage of using normal cells is that once the cells reach a superconfluent state, contact inhibition arrests any further cell proliferation. Because herpesviruses do not require the cell to be actively proliferating for completion of viral replication, we can use quiescent superconfluent HF cells to minimize the amount of cellular DNA synthesis during pulse-labeling experiments. We performed a time course experiment to determine how many days after plating were required for HF cells to reach a superconfluent state and show minimal incorporation of EdU into cellular DNA. Between days 4 and 8, cells were pulse-labeled with EdU for 30 min, followed by conjugation of the azide-coupled fluorochrome Alexa Fluor 594 to visualize incorporated EdU. There was a progressive loss of EdU-associated signal, and by days 7–8, almost no cells were incorporating EdU (Fig. S1*A*). Using these data, HF cells used for infection experiments were plated 7–8 days before pulse-labeling experiments to minimize the amount of cellular DNA synthesis. In the experiments for enriching cellular DNA, nucleoside analogue pulsing was performed in actively replicating subconfluent cells. We observed the highest EdU-associated fluorescent signal at 18 h post-plating and a decrease in signal intensity by 24 h post-plating (Fig. S1*B*). Therefore, all subsequent experiments for isolating cellular DNA in noninfected cells were performed with subconfluent cells plated 18 h before pulse-labeling experiments.

**Figure 2.**
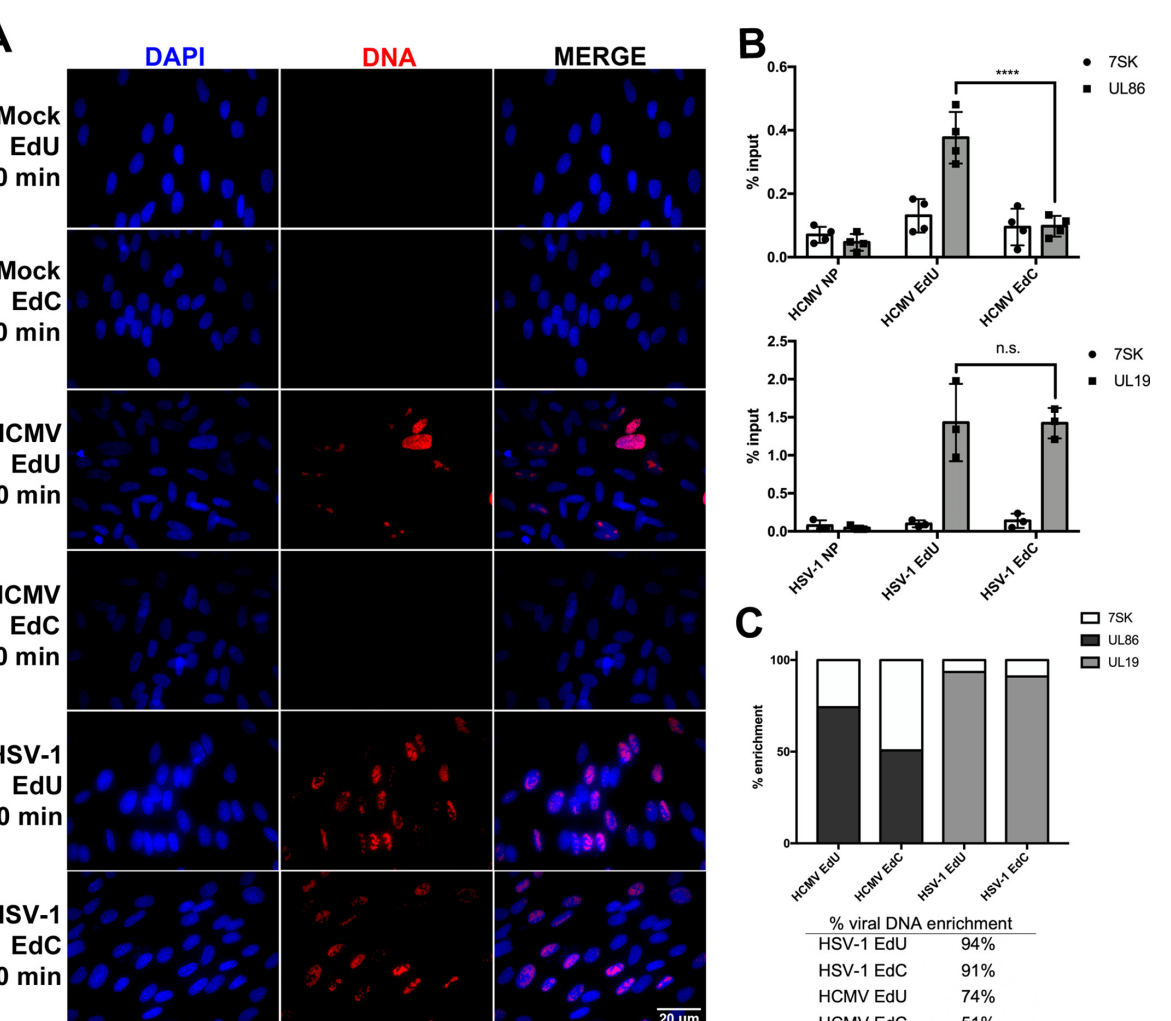
**EdC does not incorporate into nascent HCMV DNA during short time pulses.** Confluent HF cells were either mock–, HCMV AD169–, or HSV-1–infected for 72 or 6 hpi, respectively (MOI = 4). Nascent viral DNA was labeled with EdU or EdC for 30 min. *A*, infected cells were fixed, and nascent DNA was tagged with Alexa Fluor 594 (*red*). Nuclei were stained with DAPI (*blue*), and cells were imaged at ×40 magnification. *B*, labeled DNA was isolated using the FENDI protocol. Enrichment was quantified by qPCR, and percentage input was calculated. No pulse (*NP*) indicates cells that were not incubated with nucleoside analogues. *Error bars*, S.D. from three independent experiments, *n* = 3. Statistical analysis was performed using two-way ANOVA; ****, *p* < 0.0001. *C*, using the previous qPCR data to determine percentage input, percentage enrichment of viral DNA was calculated from total DNA.

Because EdC is considered a less toxic alternative to EdU, we sought to compare the labeling efficiency of both analogues (5, 31, 32). Our initial experiments for optimizing the enrichment protocol of viral DNA began by pulse-labeling HCMV– or HSV-1–infected superconfluent HF cells with EdU and EdC. To account for the differences in the length of their respective replication cycles and the onset of viral DNA synthesis, HSV-1–infected cells were pulsed at 6 h postinfection (hpi), and HCMV-infected cells were pulsed at 72 hpi. During a 30-min pulse, we were unable to visually detect EdC incorporation in HCMV-infected cells, but we were able to detect EdC incorporation in HSV-1–infected cells (Fig. 2*A*). The superconfluent HF mock-infected cells did not show any incorporation of EdC or EdU.

We quantified the relative labeling efficiency of EdU and EdC incorporation by isolating EdU- and EdC-labeled DNA from cells infected with either HSV-1 or HCMV using a technique called fast and efficient nascent DNA isolation (FENDI). After a 30-min pulse with either EdC or Edu, labeled DNA was “clicked” to biotin, sheared, and isolated using streptavidin beads. The isolated DNA was then quantified by qPCR using primers and probes for viral DNA (UL86 for HCMV and UL19 for HSV-1) and cellular DNA (7SK), and the percentage input was calculated. In agreement with the imaging in Fig. 2*A*, cells infected with HCMV had significantly less enrichment of viral DNA isolated during a 30-min pulse with EdC as compared with EdU. In contrast, cells infected with HSV-1 showed no significant difference between the enrichment of DNA labeled with EdC and EdU (Fig. 2*B*). There was no appreciable accumulation of cellular 7SK, which is consistent with previous imaging of superconfluent mock-infected cells showing no incorporation of EdC or EdU. Additionally, enrichment of EdU- or EdC-labeled viral DNA was calculated as a percentage of total enriched DNA. During an HCMV infection, viral DNA enrichment was higher during pulses with EdU (74%) than EdC (51%). In an HSV infection, viral DNA enrichment was not markedly different between EdU (94%) and EdC (91%) (Fig. 2*C*).

**Figure 3.**
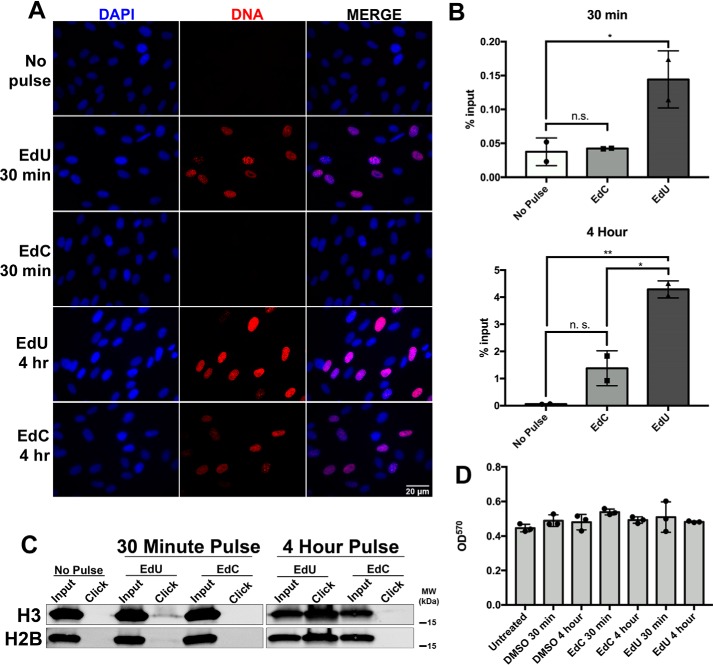
**EdC does not efficiently incorporate into nascent cellular DNA during short time pulses.** Subconfluent HF cells were labeled with EdU or EdC for 30 min or 4 h. *A*, replicating HF cells were fixed, and labeled DNA was localized using Alexa Fluor 594 (*red*), which was “clicked” to either EdU or EdC. Nuclei were stained with DAPI (*blue*), and cells were imaged at ×40 magnification. *B*, labeled DNA was isolated by the FENDI protocol. Enrichment was quantified using qPCR, and percentage input was calculated. No pulse (*NP*) indicates cells that were not incubated with nucleoside analogues. *Error bars*, S.D. from two independent experiments, *n* = 2. Statistical analysis was performed with one-way ANOVA. *, *p* < 0.05; **, *p* < 0.005. *C*, nascent cellular DNA and interacting proteins were isolated using the aniPOND protocol. Histones associated with labeled DNA were resolved by Western blotting with the indicated antibodies. *D*, cell viability assay of HF cells treated with DMSO, EdU, and EdC for 30 min or 4 h. *Error bars*, S.D. from three independent experiments, *n* = 3.

To determine whether we could visualize EdC incorporation in normal noninfected cells, pulse-labeled imaging experiments were performed using HF cells that were subconfluent. We tested EdU and EdC in HF cells that were either 50% confluent or 100% confluent. In agreement with the previous results in HCMV-infected cells, we could not visualize EdC incorporation during a 30-min pulse in subconfluent, actively replicating HF cells (Fig. S2). We next tested whether a longer pulse time (4 h) with EdC would result in incorporation. In subconfluent HF cells, we were able to detect EdC incorporation in a 4-h pulse (Fig. 3*A*). As a control, HF cells were also incubated with EdU, which showed a positive signal during the 30-min and 4-h pulse. In addition to imaging analysis, a FENDI experiment was performed to isolate nascent cellular DNA using EdC or EdU at 30 min and 4 h. The relative incorporation efficiency at each time point was determined, and percentage input was calculated by qPCR analysis using primers and probe specific to 7SK (Fig. 3*B*). During a 30-min pulse, we confirmed that EdC did not have any significant incorporation, whereas EdU showed significant enrichment. At a 4-h pulse, EdC showed a minimal amount of incorporation, whereas EdU incorporated into nascent DNA at a significantly higher level. In Fig. 3*C*, we performed an aniPOND experiment to compare the efficiency of EdU and EdC when isolating proteins associated with labeled nascent DNA. After a 30-min pulse, we detected histones (H3 and H2B) interacting with nascent DNA in HFs pulsed with EdU but not with EdC. Even during a 4-h pulse, we observed a more substantial amount of histones associated with nascent DNA in cells incubated with EdU as compared with EdC. As cytotoxicity can be a concern during these pulsing experiments, we performed an MTT cell viability assay on HF cells incubated with EdC, EdU, and vehicle (DMSO) at 30 min and 4 h (Fig. 3*D*). There was no observable cytotoxicity associated with EdC or EdU during these short time pulses.

### Efficient incorporation of EdU but not EdC after 30-min pulse-labeling in retinal pigmented epithelial (RPE) cells

**Figure 4.**
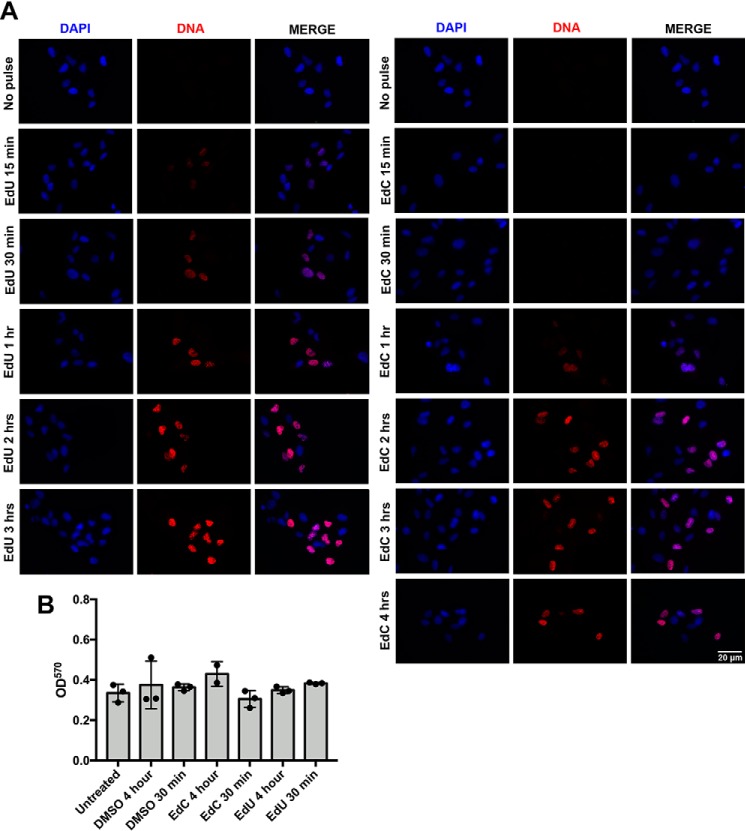
**EdU incorporation is detectable at earlier time points than EdC.** *A*, RPE cells were plated at subconfluence and labeled with EdU (*left*) or EdC (*right*) at the indicated time points. Replicating RPE cells were fixed, and labeled DNA was localized using Alexa Fluor 594 (*red*). Nuclei were stained with DAPI (*blue*), and imaging was performed under equal exposure times at ×40 magnification. *B*, cell viability assay of RPE cells treated with DMSO, EdU, and EdC for 30 min or 4 h. *Error bars*, S.D. from three independent experiments, *n* = 3.

To investigate whether the lower incorporation efficiency noted for EdC in HF cells could be extended to additional cell types, experiments were repeated in RPE cells. Because previous data demonstrated efficient EdC incorporation after longer time pulses (4 h), we performed a time course experiment by pulse-labeling cells from 15 min to 4 h. As shown in Fig. 4*A*, EdU incorporation in RPE cells can be seen after a 15-min pulse, whereas the EdC incorporation is barely visible after a 1-h pulse. An MTT assay was performed, and no cytotoxicity was associated with EdC or EdU during a 30-min and 4-h pulse in RPE cells (Fig. 4*B*).

### EdC itself does not act as a block to DNA synthesis

**Figure 5.**
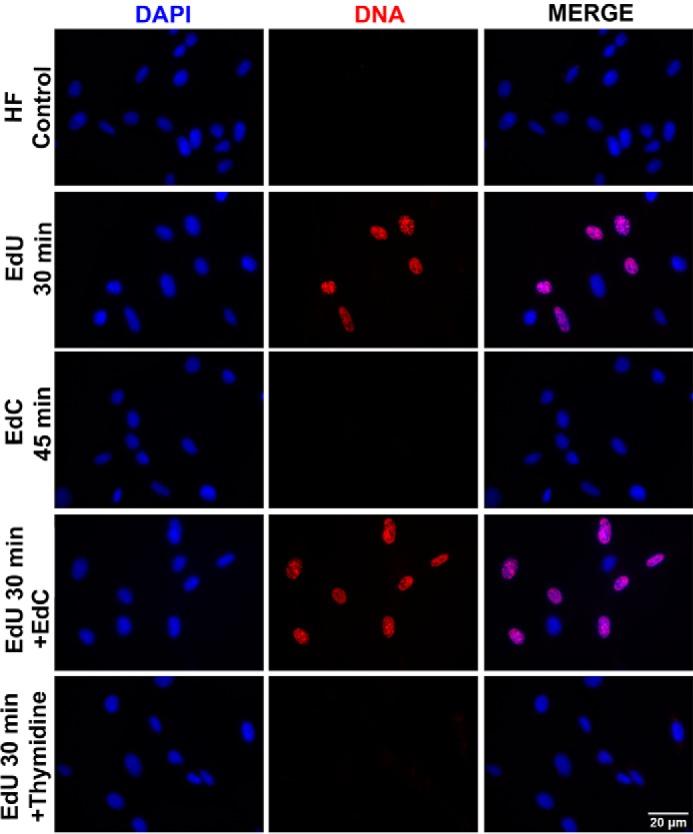
**EdC does not act as a thymidine block.** Subconfluent HF cells were incubated with EdC or Thymidine for 15 min to block DNA synthesis. After the block, HF cells were pulsed with EdU or EdC for 30 min. Cells were then fixed, and nascent DNA was labeled with Alexa Fluor 594 (*red*). Nuclei were stained with DAPI (*blue*), and imaging was performed at ×40 magnification.

An imbalance in the nucleotide pools can inhibit cellular DNA synthesis by interfering with the deoxynucleotide metabolism pathway. To rule out the possibility that the concentration of EdC (10 μm) was contributing to arrest of DNA synthesis, we pretreated cells with EdC for 15 min before the addition of EdU (10 μm) for 30 min. As a control, cells were treated with a thymidine block (10 μm), a known inhibitor of DNA synthesis, for 15 min before the addition of EdU (33, 34). The cells treated with EdC before being pulsed with EdU show levels of incorporation comparable with the cells that were only incubated with EdU. Meanwhile, cells treated with the thymidine block showed no incorporation of EdU (Fig. 5). To account for the total amount of time that the cells were incubated with EdC, a set of cells were incubated with EdC for 45 min, which showed no incorporation. Therefore, the lack of EdC incorporation during short pulses is not due to an arrest of DNA synthesis caused by a nucleotide pool imbalance.

### Efficient incorporation of EdU but not EdC after pulse-labeling KSHV-infected iSLK-BAC16 cells

Because we noted that HF cells infected with HSV-1 were able to incorporate EdC within a 30-min pulse whereas HCMV infected cells were not, we investigated whether other human herpesviruses could incorporate EdC during short pulses. We performed pulse-labeling experiments in iSLK-BAC16 cells that harbor a bacterial artificial chromosome (BAC) containing the entire Kaposi's sarcoma-associated herpesvirus (KSHV) genome. iSLK cells are an epithelial renal carcinoma cell line that have a doxycycline-inducible K-RTA, the major transactivator encoded by KSHV. These cells support a latent infection of KSHV that can be treated with doxycycline, sodium butyrate, and 12-*O*-tetradecanoylphorbol-13-acetate (TPA) to reactivate the virus (35–37).

**Figure 6.**
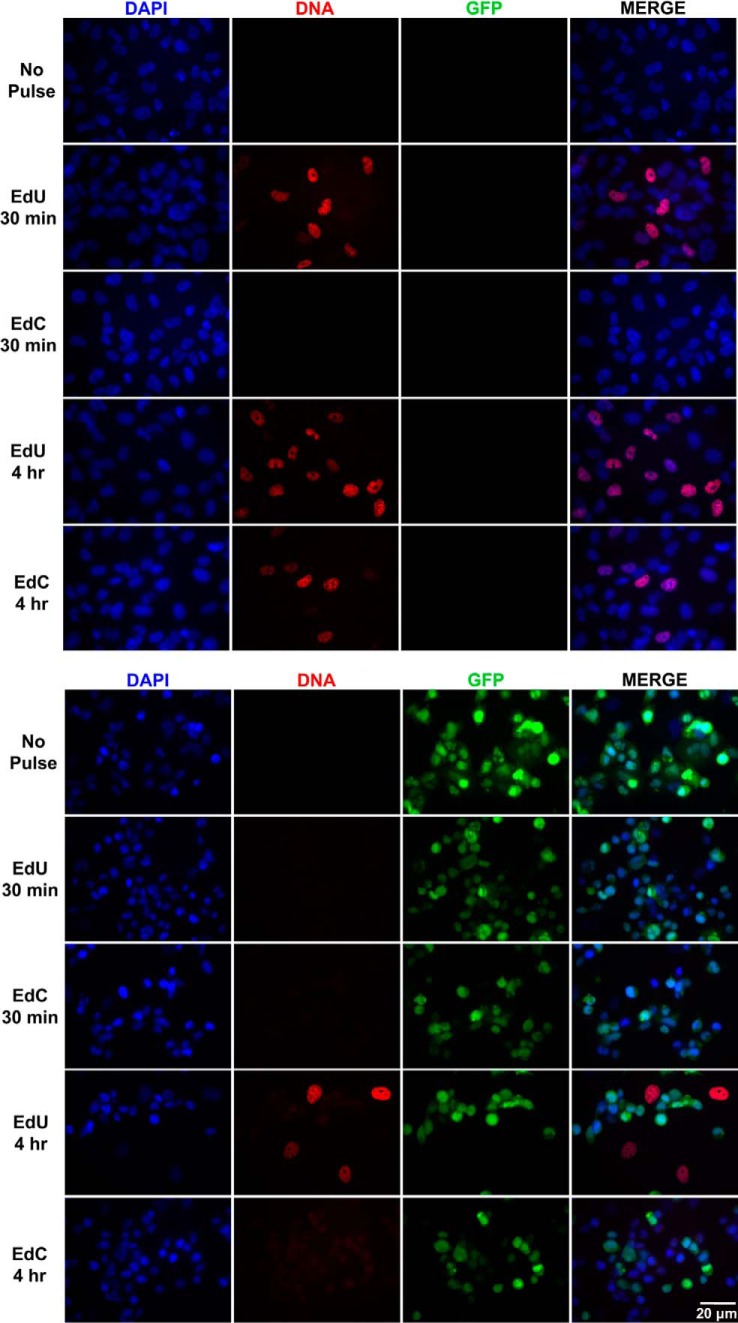
**EdC does not incorporate into nascent KSHV DNA.** iSLK (*top*) and iSLK-BAC16 (*bottom*) cells were induced with doxycycline, TPA, and sodium butyrate. KSHV BAC16 is detectable by GFP (*green*) expression. Forty-eight hours postinduction, nascent DNA was labeled with EdU or EdC for 30 min or 4 h. Cells were fixed, and labeled DNA was localized with Alexa Fluor 594 (*red*). Nuclei were stained with DAPI (*blue*). Cells were imaged at ×40 magnification under equal exposure times.

To reactivate KSHV from a latent state to an actively replicating lytic state, iSLK-BAC16 cells were treated with doxycycline, TPA, and sodium butyrate. An EdU and EdC pulse for 30 min or 4 h was performed in noninfected iSLK and iSLK-BAC16 cells during latent and lytic reactivated conditions. Shown in Fig. 6, noninfected iSLK cells have similar incorporation efficiencies as identified in HF cells and RPE cells. EdU incorporation was detectable at 30 min, whereas EdC incorporation could not be detected. At a 4-h pulse, both EdU and EdC show an incorporation signal. Interestingly, in the iSLK-BAC16 cells, there was no visible incorporation of EdC or EdU during a 30-min pulse for both the latent and reactivated conditions. However, a signal associated with EdU was detected after a 4-h pulse. This finding supports previously published work that identified that cellular DNA synthesis is disrupted during KSHV reactivation in favor of viral DNA replication (38). Because the noninfected iSLK cells did not show any differences in EdU incorporation after treatment with doxycycline, TPA, and sodium butyrate, it is doubtful that the treatment alone is causing disruption to DNA synthesis. We hypothesize that the dramatic decrease in the EdU and EdC signal in the iSLK-BAC16 is potentially due to a slower rate of nucleotide incorporation for KSHV viral polymerase. These data along with the previous experiments suggested that there is a specific viral factor that is only expressed by HSV-1, not HCMV or KSHV, which allows for the incorporation of EdC during short time pulses.

### Expression of HSV-1 TK can partially rescue the lower incorporation efficiency of EdC

Because it was observed that cells infected with HSV-1 incorporated detectable levels of EdC within a 30-min pulse, we explored what factors could be contributing to this unique phenotype. We considered viral proteins involved directly or indirectly in the deoxynucleotide metabolism pathway. Contributing to this pathway, HSV-1 has a dedicated viral TK encoded by UL23, which is capable of phosphorylating deoxypyrimidine nucleosides. In HCMV and KSHV, there are no direct genetic orthologues of UL23 (39–43). It is important to note that there are viral proteins encoded by HCMV (UL97) and KSHV (ORF36 and ORF21) that have the ability to phosphorylate antiviral nucleosides, such as ganciclovir (GCV) and acyclovir (ACV) in a similar manner to what has been reported with HSV-TK (42–45). However, HCMV UL97 and KSHV ORF36 are Ser/Thr protein kinases and do not normally phosphorylate deoxynucleosides (41, 45). The exclusive ability to phosphorylate antivirals, such as GCV and ACV, and not natural deoxynucleosides or nucleoside analogues, such as EdC, is likely due to recognition of the free hydroxyl on the acyclic side chain of GCV and ACV. Recognition of this acyclic side chain hydroxyl is similar to the recognition of the hydroxyl on the side chain of serine or threonine.

Further evidence that leads us to identify HSV-1 TK as a factor contributing to efficient EdC incorporation during short pulses arose from discussions with investigators who had performed the HSV-1 iPOND and aniPOND experiments (26–28). They observed that TK mutants of HSV-1 did not incorporate EdC and were unable to use EdC to label the replicating mutant viruses.^3^ Our next experiments were designed to determine whether HSV-TK was contributing to the ability of EdC to efficiently incorporate during short pulses.

**Figure 7.**
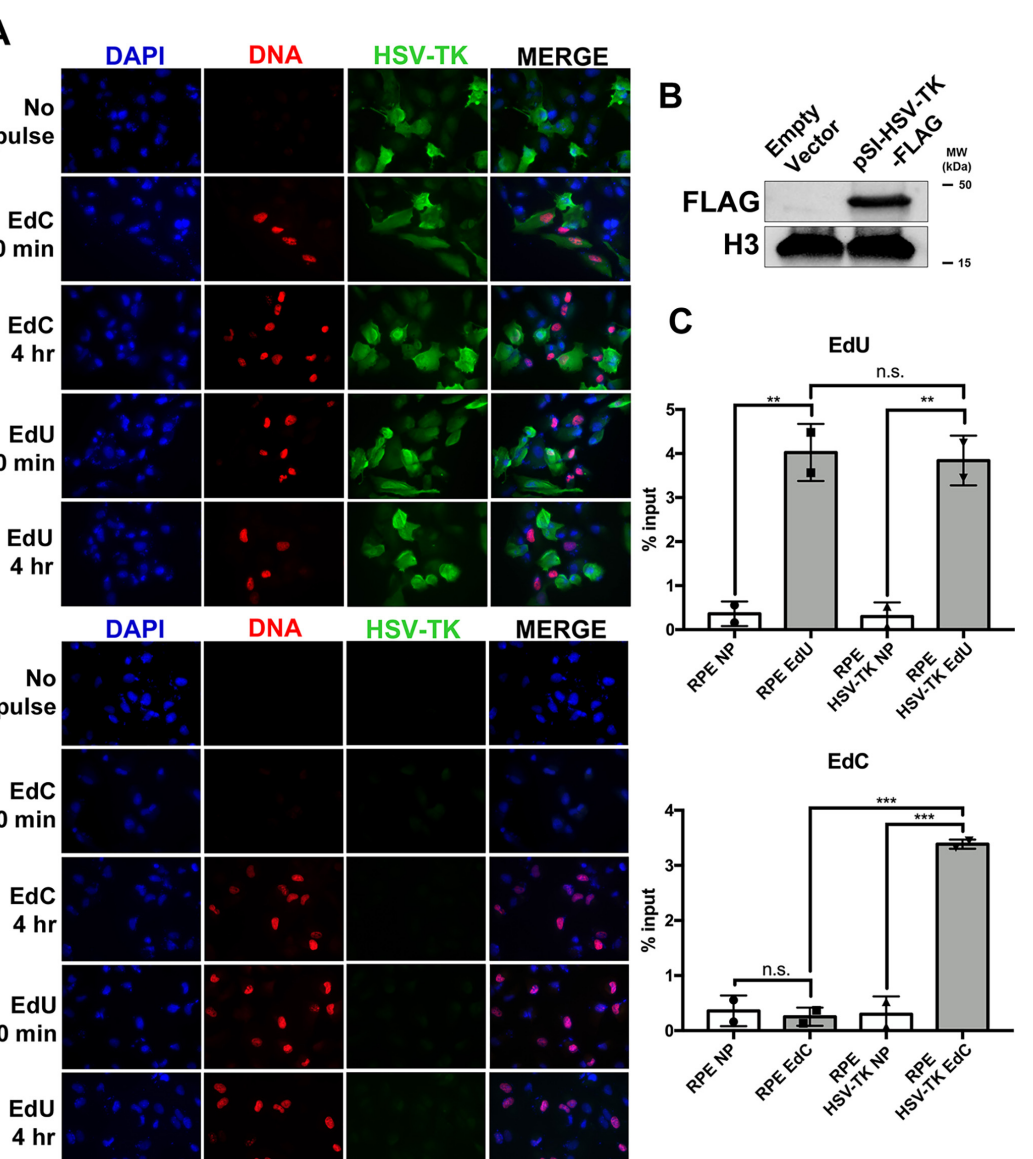
**Cells transfected with HSV-TK expression plasmid incorporate EdC during short pulses.** *A*, RPE cells were transfected with empty vector or pSI-HSV-TK-FLAG. Two days post-transfection, cells were pulsed with EdU or EdC for 30 min followed by fixation and immunofluorescent imaging. pSI-HSV-TK-FLAG was localized with Alexa Fluor 488 (*green*) and nascent DNA with Alexa Fluor 594 (*red*). Nuclei were stained with DAPI (*blue*). Imaging was performed at ×40 magnification. *B*, lysate from RPE cells transfected with empty vector or pSI-HSV-TK-FLAG was subjected to immunoblotting, and membrane was probed with the indicated antibodies. *C*, RPE cells expressing empty vector or HSV-TK were pulsed with EdU, EdC, or no pulse (*NP*). Labeled DNA was isolated with the FENDI protocol. Enrichment was quantified by qPCR, and percentage input was calculated. *Error bars*, S.D. from two independent experiments, *n* = 2. Statistical analysis was performed using one-way ANOVA. **, *p* < 0.005; ***, *p* < 0.001; *n.s.*, not significant.

We generated a series of HSV-TK expression constructs; one plasmid was created for transient expression (pSI-HSV-TK-FLAG), one to generate cell lines that stably expressed HSV-TK (pPur-EF1α-HSV-TK-GFP), and a lentivirus expression vector (pLVX-EF1α-HSV-TK-FLAG). To determine whether expression of HSV-TK influenced nucleoside analogue incorporation, we transfected RPE cells with the pSI-HSV-TK-FLAG expression plasmid or empty vector control and pulsed with EdU or EdC for 30 min or 4 h (Fig. 7*A*). Positive expression of HSV-TK was detected by immunofluorescent staining (*green*) and Western blotting (Fig. 7, *A* and *B*). In agreement with our hypothesis about HSV-TK contributing to EdC incorporation, RPE cells transfected with pSI-HSV-TK-FLAG incorporated EdC during a 30-min incubation. This was the first time EdC incorporation was detectable in noninfected and replicating cells during a short time pulse. As a control, cells transfected with empty vector and pulsed with EdC showed no appreciable signal of incorporation in the 30-min pulse. In the cells transfected with a control vector or pSI-HSV-TK-FLAG, an EdU signal was detected at 30-min and 4-h pulses in the presence and absence of HSV-TK. To verify the efficiency of pulse-labeling in the transfected cells, a FENDI experiment was performed to isolate nascent labeled DNA after a 30-min pulse. Isolated DNA was analyzed by qPCR using primers and probes to cellular 7SK. As indicated in the imaging experiments, incorporation of EdU was efficient in cells transfected with empty vector or pSI-HSV-TK-FLAG, whereas EdC incorporation was only significant in RPE cells expressing HSV-TK (Fig. 7*C*). These data identified HSV-TK as a contributing factor for EdC incorporation during short pulses.

**Figure 8.**
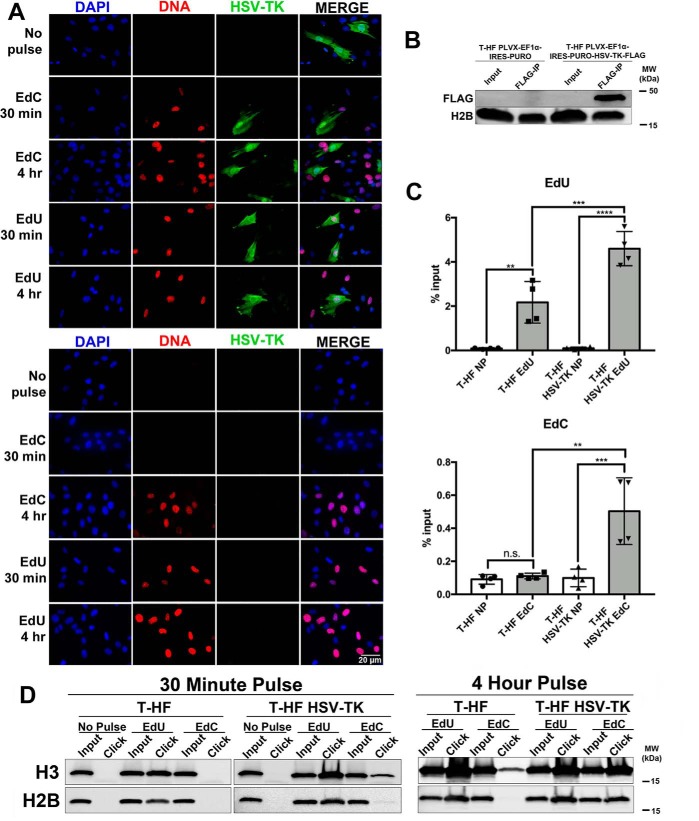
**T-HF cell line stably expressing HSV-TK-FLAG incorporate EdC during short pulses.** T-HF cells were transduced with PLVX-ef1α-IRES-Puro-HSV-TK-FLAG or PLVX-ef1α-IRES-Puro empty vector with lentivirus. Cells maintaining the constructs were selected using puromycin. *A*, T-HF HSV-TK-FLAG (*top*) and T-HF empty vector (*bottom*) cells were pulsed with EdU and EdC for 30 min or 4 h. Cells were fixed, and immunofluorescent imaging was performed. Expression of HSV-TK-FLAG was visualized with Alexa Fluor 488 (*green*), nascent DNA was labeled with Alexa Fluor 594 (*red*), and nuclei were stained with DAPI (*blue*). Imaging was performed at ×40 magnification. *B*, cell lysates were harvested and subjected to immunoprecipitation followed by Western blotting for detection of HSV-TK-FLAG. Membrane was probed with antibody as indicated. *C*, labeled nascent DNA was isolated using the FENDI protocol. Enrichment of DNA was quantified by qPCR, and percentage input was calculated. *NP*, no pulse. *Error bars*, S.D. from four independent experiments, *n* = 4. Statistical analysis was performed using one-way ANOVA. **, *p* < 0.005; ***, *p* < 0.001; ****, *p* < 0.0001; *n.s.*, not significant. *D*, proteins associated with nascent cellular DNA were isolated following the aniPOND protocol and resolved by Western blot analysis. Membrane was probed with the indicated histone antibodies.

Next, we generated cell lines expressing HSV-TK to test EdC incorporation efficiency. The first cell line was made using life-expanded telomerized human fibroblasts (T-HFs), which were transduced with lentivirus expressing a pLVX-Ef1α-HSV-TK-FLAG and selected for puromycin resistance. Expression of HSV-TK-FLAG in the T-HF HSV-TK cell line was detected by immunofluorescent imaging (*green*) and Western blot analysis (Fig. 8, *A* and *B*). Corresponding to our previous results with RPE cells transiently expressing HSV-TK-FLAG, only the T-HF cells that expressed HSV-TK were able to efficiently incorporate EdC in a 30-min pulse, whereas the T-HF control cells show no incorporation of EdC in a 30-min pulse (Fig. 8*A*). We further verified incorporation efficiency of EdU and EdC during a 30-min pulse using a FENDI experiment to measure enrichment of labeled DNA. Similar to previous experiments, EdU incorporation was significant in T-HF and T-HF HSV-TK cell lines, whereas EdC enrichment was only significant in cells expressing HSV-TK (Fig. 8*C*). To investigate proteins associating with labeled nascent DNA in our HSV-TK–expressing cell line, an aniPOND experiment was performed with cells pulse-labeled with EdU or EdC for 30 min or 4 h. In a 30-min pulse, histones associated with EdU-labeled DNA were detectable in both cell lines, whereas histones associated with EdC-labeled DNA were only detected in the presence of HSV-TK (Fig. 8*D*). During a 4-h pulse, T-HF HSV-TK cells showed comparable enrichment of associated histones between the EdU and EdC pulse.

**Figure 9.**
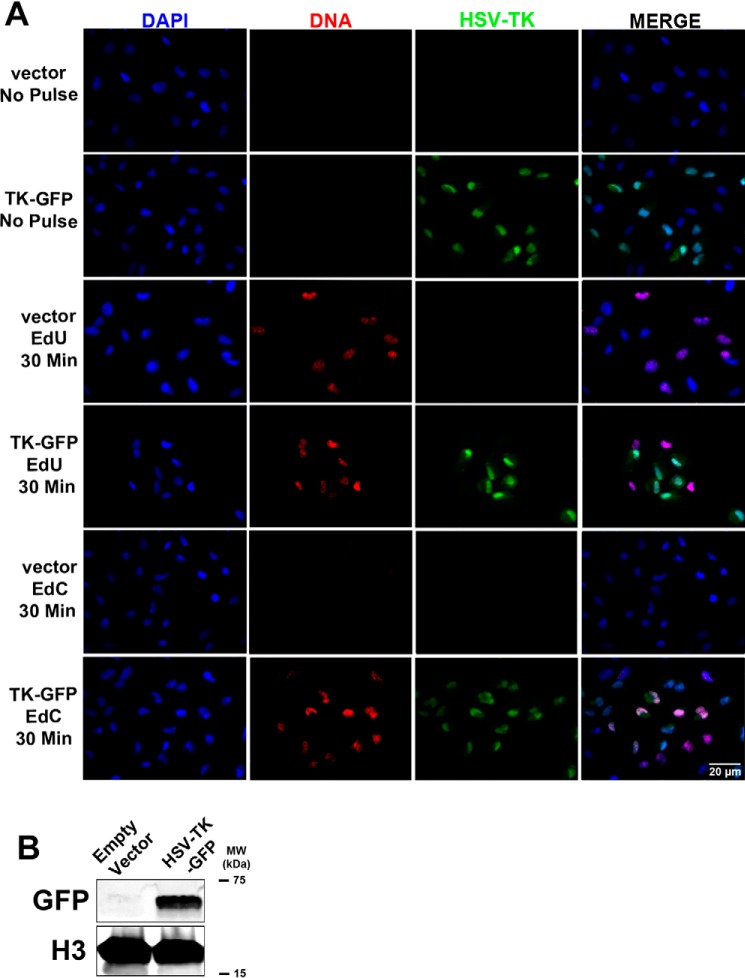
**RPE cell line stably expressing HSV-TK-GFP incorporate EdC during short pulses.** RPE cells were transfected with empty vector or pPur-EF1α-intron-MCS-TKpolyA-HSV-TK-GFP. Cells maintaining the transfected constructs were selected for using puromycin. *A*, RPE empty vector and RPE HSV-TK-GFP were pulsed with EdU or EdC for 30 min followed by fixation. Immunofluorescent imaging was used to detect HSV-TK-GFP (*green*), nascent DNA conjugated to Alexa Fluor 594 (*red*), and nuclei stained with DAPI (*blue*) at ×40 magnification. *B*, lysates were subjected to Western blotting to detect HSV-TK-GFP expression, and membrane was probed with the indicated antibodies.

A second cell line was created using RPE cells transfected with pPur-EF1α-TK-GFP or an empty vector plasmid and selected for puromycin resistance. Expression of HSV-TK-GFP was verified by immunofluorescent imaging and Western blot analysis (Fig. 9, *A* and *B*). Similar to T-HF expressing HSV-TK, RPE HSV-TK-GFP cells were able to incorporate EdC in a 30-min pulse, whereas cells that contained the vector control were unable to incorporate EdC in a 30-min pulse (Fig. 9*A*). These data collectively demonstrate the requirement of HSV-TK expression for efficient EdC incorporation during short time pulses.

### Treatment with zebularine, an inhibitor of cytidine deaminase, can prevent incorporation of EdC but not in the presence of HSV-TK

**Figure 10.**
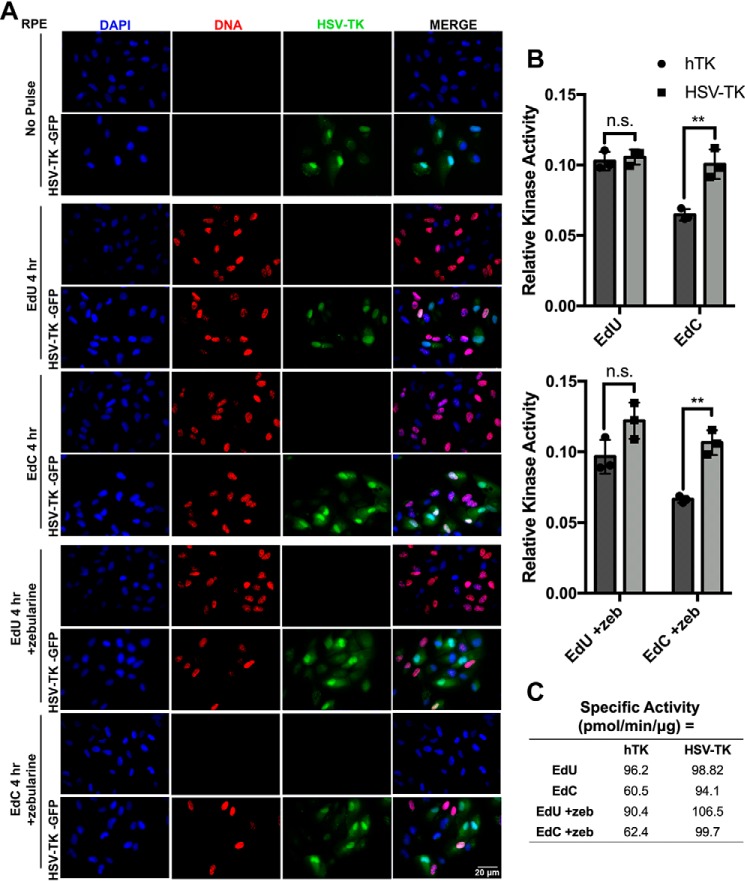
**Inhibiting cytidine deaminase does not inhibit EdC incorporation in the presence of HSV-TK.** *A*, RPE cells expressing pPur-EF1α-intron-MCS-TKpolyA-HSV-TK-GFP or empty vector were untreated or treated with zebularine. Cells were then pulsed with EdU or EdC for 30 min or 4 h. Immunofluorescent imaging was performed to detect expression of HSV-TK-GFP (*green*) and nascent DNA conjugated to Alexa Fluor 594 (*red*). Nuclei were stained with DAPI (*blue*). Images were taken at ×40 magnification. *B*, a protein kinase A assay was used to determine phosphorylation activity of HSV-TK and hTK on 2-deoxycytidine, thymidine, EdC, and EdU (*top*). The kinase assay was repeated in the presence of zebularine (*bottom*). *Error bars*, S.D. from three independent experiments, *n* = 3. Statistical analysis was performed using two-way ANOVA. **, *p* < 0.005; *n.s.*, not significant. *C*, calculation of specific activity of HSV-TK and hTK.

Cytidine deaminase catalyzes the conversion of cytidine and deoxycytidine to uridine and deoxyuridine, respectively. EdC can be readily converted into EdU due to deamination of EdC by cellular cytidine deaminases, and it was previously reported that the majority of incorporated EdC is in fact EdU (24). To further explore the potential conversion of EdC to EdU in the presence of HSV-TK, we treated cells with zebularine, a potent inhibitor of cytidine deaminase. Zebularine mimics the structure of cytidine and binds to cytidine deaminase to block the interaction to its natural substrate (46). RPE and RPE HSV-TK-GFP cells were untreated or treated with 500 μm zebularine before pulse-labeling with EdU or EdC for 4 h (Fig. 10*A*). As expected, the untreated cells showed efficient incorporation of EdU and EdC. In the presence of zebularine, RPE cells pulsed with EdU showed no significant incorporation deficiencies, but cells pulsed with EdC showed significant defects in incorporation. Interestingly, the RPE HSV-TK-GFP cells treated with zebularine showed incorporation of EdC (Fig. 10*A*). This suggested that HSV-TK is able to phosphorylate EdC, ultimately leading to incorporation into nascent DNA without EdC first being deaminated into EdU.

Because cells treated with zebularine were only able to incorporate EdC in the presence of HSV-TK expression, we hypothesized that EdC was phosphorylated by HSV-TK at a higher rate than cellular kinases. To further investigate potential differences in the rate of phosphorylation of EdU and EdC, we performed an *in vitro* kinase assay with HSV-TK and human thymidine kinase (hTK) and determined the relative rates of phosphorylation activity. HSV-TK and hTK enzymes are responsible for the rate-limiting step in the pathway to produce nucleosides in their triphosphate forms. HSV-TK has been described as both a thymidine kinase and deoxycytidine kinase (47, 48). The use of hTK in these experiments is for relative comparison, as hTK is a cellular homologue of HSV-TK. A nonradioactive universal kinase assay was performed with purified hTK and HSV-TK using either EdU or EdC as the phosphate acceptor. The kinases transfer the terminal phosphate group from ATP to an acceptor substrate, in this case either EdU or EdC, which produces ADP as a by-product of the reaction. The reaction mixture also contains CD39L2 phosphatase to release the β-phosphate from the newly created ADP (49). The P_i_ released from the ADP is proportional to the amount of ADP generated during the kinase reaction and reflects the kinetics of the kinases (hTK or HSV-TK) with the acceptor substrate (EdU or EdC). Malachite green reagents were used to detect the phosphate released from ADP. A standard curve of known ADP concentrations was generated to calculate the coupling rate specific activity of the kinase reaction on EdU or EdC after a single 20-min incubation. There was no difference in the relative rate of phosphorylation with hTK and HSV-TK on EdU, but for EdC, the rate of phosphorylation was significantly lower with hTK compared with HSV-TK (Fig. 10*B*). Specific activity of hTK is significantly lower for EdC (60.5 pmol/min/μg) than EdU (96.2 pmol/min/μg) (Fig. 10*C*). However, HSV-TK has similar specific activity for both EdC (94.1 pmol/min/μg) and EdU (98.82 pmol/min/μg). The kinase assay was also performed in the presence of zebularine (+*zeb*) (Fig. 10, *B* and *C*). The addition of zebularine did not affect the rate of kinase activity for either hTK or HSV-TK.

**Figure 11.**
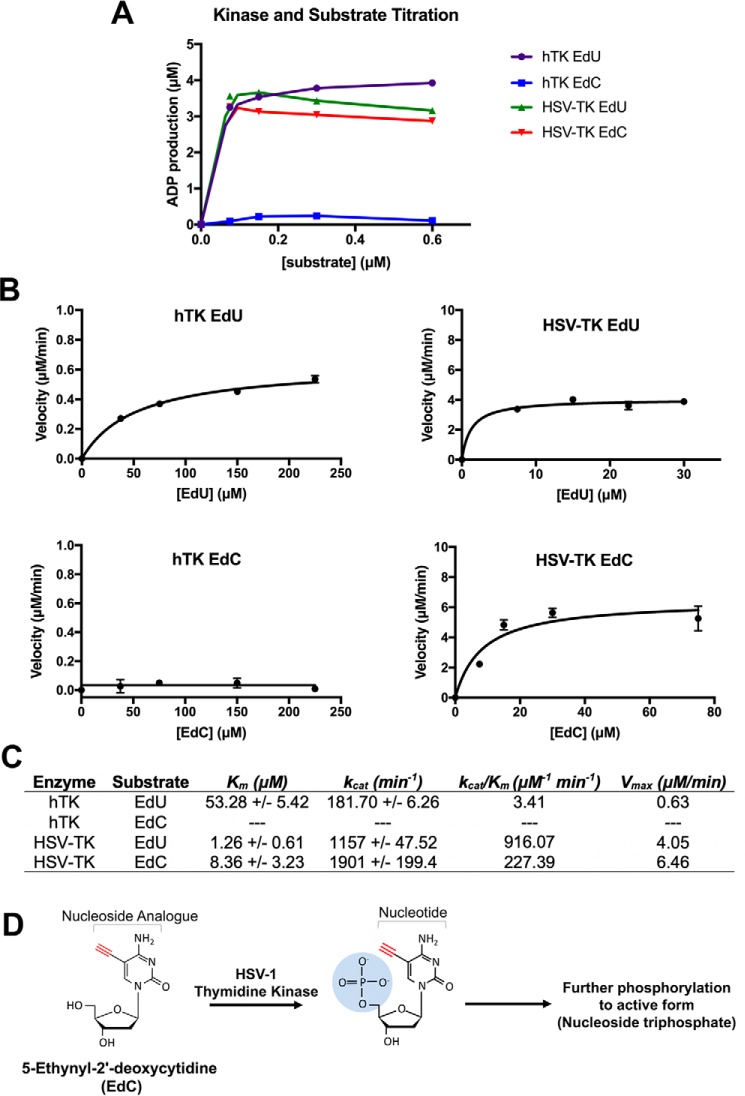
**HSV-1 thymidine kinase phosphorylates EdC.** *A*, titration curve of hTK or HSV-TK phosphorylation activity in the presence of increasing concentrations of EdU or EdC. *B*, Michaelis–Menten plots of enzymatic reaction rate of EdU or EdC in the presence of hTK or HSV-TK. Phosphorylation activity was measured by ADP production, and enzymatic activity was fitted to Michaelis–Menten parameters. *Error bars*, S.D. from three independent experiments, *n* = 3. *C*, *table* summarizing catalytic efficiency calculated from rates fitted to Michaelis–Menten curves. *D*, *schematic* showing that EdC is phosphorylated by HSV-1 thymidine kinase. HSV-1 thymidine kinase catalyzes the addition of the α-phosphate (highlighted in *blue*), therefore priming the nucleotide to undergo further phosphorylation into its active form (nucleoside triphosphate).

To further assess the enzymatic characteristics of HSV-TK and hTK with EdU and EdC, enzyme kinetic studies were performed. Edu and EdC substrate titration curves were calculated with HSV-TK and hTK by measuring the concentrations of ADP byproduct because ATP is used as the phosphate donor during the kinase reaction (Fig. 11*A*). Based on the substrate titration measurements, substrate concentrations within the linear range were used in the subsequent enzyme kinetic experiments. Michaelis–Menten curves were generated by varying the concentration of substrate over time and measuring the concentration of ADP produced (Fig. 11*B*). Using these measurements, the *K_m_* and *k*_cat_ values were calculated for hTK and HSV-TK with EdU and EdC. The *K_m_* for HSV-TK with EdU is 1.26 ± 0.61, and with EdC it is 8.36 ± 3.23. The *K_m_* for hTK with EdU is 53.28 ± 5.42, and with EdC we were unable to calculate the *K_m_*. The *k*_cat_ for hTK with EdU is 181.70 ± 6.26, HSV-TK with EdU is 1157 ± 47.52, and HSV-Tk with EdC is 1901 ± 199.4 (Fig. 11*C*). The inability to accurately measure and report a *K_m_* and *k*_cat_ value for hTK with EdC in this assay demonstrates that hTK cannot efficiently phosphorylate EdC.

These findings suggest that the phosphorylation activity of HSV-TK on EdC ultimately leads to its efficient incorporation into nascent DNA (Fig. 11*D*). Therefore, pulse-labeling experiments using EdC require the expression of HSV-TK to enhance EdC incorporation during short time pulses.

## Discussion

Nucleoside analogues are a powerful tool in molecular biology. Over the years there have been many variations of nucleoside analogues. Different bases have been used with various additions or side groups, which have allowed for diverse types of downstream applications. Some of the very first nucleoside analogues used to detect the newly synthesized DNA and subsequent cell proliferation were radiolabeled (3, 50). The additional safety procedures that go along with using radioisotopes have necessitated the development of nucleoside analogues that can be detected without using radioactivity; most notable is BrdU, which can be detected with a specific antibody (4, 51). There are challenges using BrdU because the detection method is antibody-based, which requires the cells to be fixed, permeabilized, and denatured for the antibody to have access to the intracellular BrdU epitope. Newer methods use nucleoside analogues that contain an alkyne modification, such as EdU or EdC, followed by click chemistry to attach an azide containing fluorescent dye or biotin molecule (5, 6, 52). Due to the small size of these reagents, they readily enter the cell and access the DNA without the need for harsh treatments associated with previous generations of nucleoside analogues. This leads to protocols that are simpler and faster and can be multiplexed with other reagents for immunofluorescent assays or quantitative DNA analysis.

One of the interesting things about the nucleoside analogues used to label DNA is that they are often derivatives of 2′-deoxyuridine or thymidine. The potential issue regarding the use of 2′-deoxyuridine analogues is cytotoxicity during long-term labeling experiments leading to cell cycle arrest and cell death. Two prevailing causes for the cytotoxicity are the uracil-associated mismatch or transition mutation in DNA; this results in activation of the DNA repair pathway, which recognizes and replaces the uracil (53). The reason for the development and availability of the 2′-deoxyuridine analogues is partly due to their original use as potential chemotherapeutics or antivirals (12–14, 54–57). Also, the structure of 2′-deoxyuridine allows for the hydrogen at the 5-position of uracil to be readily replaced with modifications, such as the ethynyl group in EdU. It would seem like a solution to the problems associated with EdU and EdC would be to use purine analogues. Whereas there are reports of the use of ethynyl-modified purine analogues, such as 7-deaza-7-ethynyl-2′-deoxyadenosine (EdA) or 7-deaza-7-ethynyl-2′-deoxyguanosine (EdG), for DNA labeling, these analogues are not widely commercially available (58, 59). This is partly due to the difficultly in making substitutions to the purine bases, usually at the 7-position, which require a more challenging multistep synthesis process compared with the 5-position–substituted uracil analogues (60, 61).

We initially sought to determine the optimal conditions for pulse-label experiments with nucleoside analogues that would provide the highest enrichment of labeled viral DNA while minimizing labeled cellular DNA during an infection with HCMV. During our efforts to optimize the protocol, we determined that there was varying efficiency in the incorporation of EdU and EdC. Data collected from fluorescent imaging clearly demonstrated that there was a defect in the incorporation of EdC compared with EdU. These data were confirmed by qPCR and Western blot analysis, which showed that there was a lower enrichment of EdC-labeled DNA relative to EdU-labeled DNA. To rule out the possibility that enrichment was cell type–specific, we performed EdU and EdC pulse-labeling experiments in normal human fibroblasts, telomerized HF, RPE, and iSLK cells. These cells represented normal, transformed, and life-expanded cell types, and all showed a consistent lower incorporation of EdC compared with EdU.

Because we were interested in using the analogues to enrich nascent viral DNA, we performed the EdC and EdU incorporation comparison in cells infected with HSV-1, HCMV, and KSHV. These viruses are representative members of the three subfamilies of human herpesvirues; HSV-1 is an alphaherpesvirus, HCMV is a betaherpesivirus, and KSHV is a gammaherpesvirus. Surprisingly, HSV-1 showed a comparable level of incorporation of both EdC and EdU, whereas HCMV, KSHV, and noninfected cells showed lower incorporation of EdC compared with EdU. These observations along with personal correspondence with Dr. Jill Dembowski^3^ led us to speculate that HSV-TK, which does not have a direct homologue in HCMV or KSHV, was allowing for the efficient incorporation of EdC. To test this hypothesis, we created plasmids and cell lines that expressed HSV-TK and found a significant increase in EdC incorporation in the presence of HSV-TK. These results were consistent in cells that transiently expressed HSV-TK as well as cell lines that stably expressed HSV-TK. To quantitate the specific phosphorylation rates, we performed an *in vitro* kinase assay with hTK and HSV-TK using EdC and EdU as the phosphate acceptor substrate. The rate of phosphorylation of EdC was significantly lower for hTK compared with HSV-TK, whereas the rates of phosphorylation of EdU for hTK and HSV-TK were similar. We cannot rule out that there is another cellular kinase, most likely deoxycytidine kinase, that would phosphorylate EdC more readily than hTK. It remains to be seen how the physiological concentrations and activity of hTK and HSV-TK *in vivo* correlate to the *in vitro* kinase activity that we report here. We cannot exclude the possibility that a higher concentration of cellular kinase, not specifically HSV-TK, would also contribute to an increase in EdC incorporation. However, our imaging, FENDI, and aniPOND data clearly showed a defect in EdC incorporation, which was overcome through expression of HSV-TK. This suggested that lower EdC incorporation is due to an inability of cellular deoxypyrimidine kinases to phosphorylate EdC during short time pulses (<30 min).

EdC is considered to be a less toxic alternative to EdU, and it was unexpected to find the incorporation of EdC into nascent DNA to be significantly lower than EdU. This was particularly surprising during the infection studies with HCMV and KSHV, as these viruses (along with most herpesviruses) encode proteins that normally prevent the incorporation of deoxyuridine into DNA to preserve fidelity. The virus encodes factors to prevent this incorporation by decreasing the pools of dUTP either through the activity of viral dUTPases or through recognition of incorporated deoxyuridine by viral UNG enzymes (62–64). Whereas many viral dUTPases or vUNG are dispensable in normal cell culture models of infection, these viral enzymes are required for viral replication and pathogenesis for *in vivo* models of infection (65–70). Labeling with EdU for longer time pulses, especially when creating labeled infectious virions, is reported to decrease titers in cells incubated with EdU (26, 71). For HSV-1, this decrease can be overcome by deletion of the viral dUTPase (UL50) and vUNG (UL2) (26). Given the activity of these enzymes to recognize and decrease deoxyuridine, we demonstrate that EdU is still readily incorporated into viral DNA during short time pulses without any associated cytotoxicity.

The use of EdU during longer pulsing experiments warrants careful consideration of alternative nucleoside analogues because of reported cytotoxicity associated with EdU (17). The data presented here clearly demonstrate that during short pulsing experiments, EdU has a higher incorporation compared with EdC as well as no increase in cytotoxicity. The imaging results, along with qPCR from labeled nascent DNA and Western blotting from aniPOND all support the conclusion that there is significantly less EdC incorporated into DNA during short time pulses. This establishes EdU as the preferable nucleoside analogue for increased enrichment of labeled nascent DNA. We also reported the experimental conditions necessary to provide a high specific enrichment of viral DNA while minimizing cellular DNA, resulting in a 94% enrichment of HSV-1 DNA and 74% enrichment of HCMV DNA in the total labeled DNA recovered. We demonstrated that transient expression HSV-TK or HSV-TK cell lines can be used in conjunction with EdC as an alternative to EdU for researchers who are concerned with EdU-associated cytotoxicity while providing efficient incorporation of EdC.

## Experimental procedures

### Cells and virus

HFs, T-HFs, RPE (ARPE-19 ATCC), 293FT, RPE HSV-TK-GFP, T-HF PLVX-ef1α-IRES-Puro, and T-HF PLVX-ef1α-IRES-Puro-HSV-TK-FLAG cells were maintained in Dulbecco's modified Eagle's medium supplemented with 10% fetal bovine serum (Corning) and grown at 37 °C in 5% CO_2_. HCMV AD169 and HSV-1 KOS (ATCC) viral stocks were grown on HF cells, and viral titers were measured by a plaque assay.

### Plasmids

To generate the pSI-HSV-TK-FLAG construct, pSI-SV40 (Promega) was linearized using enzymes MluI and XbaI. A FLAG-tagged HSV thymidine kinase DNA fragment (HSV-TK FLAG) was synthesized as a gBlock (IDT) (Table S1). HSV-TK-FLAG was cloned into pSI-SV40 using GeneArt Seamless Cloning and Assembly (Thermo Fisher Scientific) followed by transformation into One Shot® TOP10 *Escherichia coli* (Thermo Fisher Scientific). For the PLVX-ef1α-IRES-Puro-HSV-TK-FLAG construct, the pLVX-ef1α-IRES-Puro vector (Clontech) was linearized with BamHI and EcoRI, and the HSV-TK-FLAG gBlock (IDT) was cloned into the vector and transformed as described previously. To construct a pPur-EF1α-intron-MCS-TKpolyA vector, pPUR (Clontech) was linearized using the enzymes BamHI and EcoRI. EF1α was PCR-amplified from pLVX-ef1α-IRES-Puro, and a gBlock was used to synthesize the intron-MCS-TKpolyA DNA region (IDT) (Table S1). EF1α and intron-MCS-TKpolyA were cloned into pPUR in one reaction and transformed as described previously. For the pPur-EF1α-intron-MCS-TKpolyA-HSV-TK-GFP construct, pPur-EF1α-intron-MCS-TKpolyA was linearized using BamHI and EcoRI. HSV-TK was PCR-amplified from pSI-HSV-TK-FLAG, and GFP was amplified from pEGFP-N1 (Clontech) (Table S1). HSV-TK–tagged GFP was cloned into pPur-intron-MCS-TKpolyA in a single reaction as described previously.

### Cell lines expressing HSV-TK

For the T-HF cell line expressing pLVX-ef1α-IRES-Puro-HSV-TK-FLAG, 293FT cells were transduced with pLVX-ef1α-IRES-Puro-HSV-TK-FLAG or pLVX-ef1α-IRES-Puro empty vector using the Lenti-X Packaging Single Shots system (VSV-G) (Clontech). Two days post-transduction, lentivirus was harvested and filtered through a 0.45-μm filter. T-HFs were then infected with the lentivirus containing either pLVX-ef1α-IRES-Puro-HSV-TK-FLAG or pLVX-ef1α-IRES-Puro empty vector. Three days postinfection, cells maintaining constructs were selected for at 0.25–0.5 μg/ml puromycin. For the RPE cell line expressing pPur-intron-MCS-TKpolyA-HSV-TK-GFP, RPE cells were transfected with pPur-intron-MCS-TKpolyA-HSV-TK-GFP using TransIT-LT1 transfection agent (Mirus) at a 2:1 DNA to transfection agent ratio. Two days post transfection, cells maintaining the HSV-TK-GFP construct were selected for using 0.25–0.5 μg/ml puromycin.

### Western blot analysis and co-immunoprecipitation

To verify expression of pSI-HSV-TK-FLAG, 1 × 10^6^ RPE cells were transfected with 3 μg of pGEM or pSI-HSV-TK-FLAG using TransIT-LT1 transfection agent as described previously. Two days post-transfection, cells were washed with PBS and lysed using radioimmune precipitation assay buffer (Thermo Fisher Scientific) and protease inhibitor 1:100 (Millipore Sigma). Samples were sheared by sonication, and debris was pelleted at 13,000 rpm for 10 min. The supernatant was collected, and protein was denatured using Laemmli buffer with β-mercaptoethanol and heated at 95 °C for 5 min. Protein was resolved by SDS-PAGE (Bio-Rad) and transferred to a nitrocellulose membrane (Bio-Rad). The membrane was blocked using 5% BSA in TBST (TBS, 0.01% Tween 20) or Odyssey Blocking Buffer (LI-COR). Primary antibodies were then diluted in blocking buffer, and membrane was incubated overnight at 4 °C with FLAG Ab 1:1000 (Millipore Sigma) followed by Alexa Fluor rabbit Ab 680 1:10,000 (Thermo Fisher Scientific) and visualized using LI-COR Odyssey or Bio-Rad ChemiDoc MP imaging systems. The membrane was reprobed with H3 Ab 1:5000 (Abcam, catalogue no. 1791) for 1 h at room temperature followed by Alexa Fluor rabbit Ab 800 1:10,000 (Thermo Fisher Scientific). To determine expression of HSV-TK-FLAG from T-HF PLVX-ef1α-IRES-Puro-HSV-TK-FLAG or T-HF PLVX-ef1α-IRES-Puro empty vector, supernatant was collected as described previously and incubated with anti-FLAG M2 magnetic beads (Millipore Sigma) overnight, or a fraction was saved for input. The next day, magnetic beads were washed three times with 1× TBS, and protein was eluted by boiling in Laemmli buffer with β-mercaptoethanol. Western blot analysis was performed as described above. After blocking, the membrane was probed with H2B Ab 1:2000 (Abcam, catalogue no. 52484) and reprobed with FLAG Ab, and protein was detected with Alexa Fluor mouse Ab 680. For detection of HSV-TK expression in RPE pPur-EF1α-intron-MCS-TKpolyA-HSV-TK-GFP, supernatant was collected, and Western blot analysis was performed as described previously. After blocking, the membrane was probed with GFP Ab 1:5000 (Abcam, catalogue no. 13970) overnight at 4 °C, followed by Alexa Fluor chicken Ab 800 1:10,000 (Thermo Fisher Scientific). Membrane was reprobed with H3 Ab. AniPOND experiments were performed as described, and proteins associating with nascent DNA were analyzed by Western blotting. Membranes were probed with H3 Ab and H2B Ab.

### Immunofluorescent imaging

Confluent HF cells on coverslips in a 12-well plate were infected with AD169 or HSV-KOS (MOI = 4) and pulsed with 10 μm EdU or EdC for 30 min at 72 and 6 hpi, respectively. Medium was removed, and cells were washed once with PBS, followed by fixation using 4% paraformaldehyde for 15 min at room temperature. Cells were then permeabilized with 0.5% Triton X-100 in PBS for 20 min at room temperature. The Click-It Plus Alexa Fluor 594 imaging kit (Thermo Fisher Scientific, catalog no. C10693) was used according to the manufacturer's protocol to visualize nucleoside analogue incorporation into nascent DNA. After the click reaction, coverslips were mounted onto glass slides using ProLong Gold Antifade reagent with DAPI (Invitrogen). Slides were cured for 24 h before imaging with a fluorescent microscope (Carl Zeiss, Inc.). HF, T-HF, and T-HF HSV-TK-FLAG cells, RPE cells, and RPE HSV-TK-GFP cells were plated on coverslips at subconfluence (50,000 cells/well) or grown to superconfluence. Cells were pulsed with 10 μm EdU or EdC for 30 min or 4 h. Fixation, permeabilization, and click reaction was performed as described above. Cells were mounted and imaged. RPE cells were seeded on coverslips and transfected the next day with 1 μg of pSI-HSV-TK-FLAG or pGEM DNA using the TransIT-LT1 transfection agent (Mirus). Two days post-transfection, cells were incubated with 10 μm EdC or EdU for 30 min. Cells were fixed and permeabilized, and a click reaction was performed as described above. After the click reaction, cells were washed and blocked for 30 min with 3% goat serum in PBS at room temperature. FLAG antibody (Millipore Sigma F3165) was diluted in 3% BSA in PBS (1:1000) and incubated at room temperature for 2 h to label FLAG protein. Cells were washed three times in PBS with 0.5% Tween and incubated with Alexa Fluor 488 (1:1000) for 30 min at room temperature. Secondary antibody was removed, and cells were washed with 3% BSA in PBS three times followed by mounting onto glass slides as described. iSLK and iSLK BAC16 cells were plated on coverslips at 75,000 cells/well. The next day, cells were induced with 0.5 mm sodium butyrate (Millipore Sigma), 0.5 μg/μl doxycycline (US Biologicals), and 10 ng/ml TPA (Millipore Sigma). After 48 h postinduction, cells were pulsed and imaged as described. Subconfluent HFs were incubated with EdC (20 μm) or thymidine (4 mm) for 15 min to block DNA synthesis. HFs were then pulsed with 10 μm EdU or EdC for 30 min. Cells were mounted and imaged as described previously. RPE cells and RPE cells expressing HSV-TK-GFP were plated at subconfluence on coverslips. The next day, cells were treated with 500 μm zebularine (Millipore Sigma) for 45 min prior to pulsing with 10 μm EdU or EdC for 4 h. Pulsing medium also contained zebularine to maintain initial treatment concentration. After pulsing, cells were fixed and permeabilized, and a click reaction was performed as described.

### Cell viability assay

HF and RPE cells were plated at 5000 cells/well in a 96-well plate. Cells were pulsed with 10 μm EdU or EdC or with DMSO for either 30 min or 4 h. The Cell Titer 96 Non-Radioactive Cell Proliferation assay (Promega) was used to determine cell toxicity.

### FENDI

The following method was adapted from the protocol of Dembowski and Deluca (27). Subconfluent HF, T-HF, or T-HF HSV-TK-FLAG cells (1,000,000 cells) or HF cells infected with AD169 or HSV-KOS (10-cm dish), were pulsed with prewarmed medium containing 10 μm EdU or EdC for 30 min or 4 h. To stop the pulse, cells were washed once with ice-cold PBS followed by adding 1 ml of cold nucleus extraction buffer (NEB) (20 mm Hepes, pH 7.2, 50 mm NaCl, 3 mm MgCl_2_, 300 mm sucrose, and 0.5% IGEPAL CA630) for 20 min. Cells were scraped into a tube, and nuclei were pelleted by centrifugation at 800 RCF for 10 min at 4 °C. The nucleus pellet was washed with ice-cold PBS and centrifuged at 800 RCF for 10 min at 4 °C. A click reaction was made using the Click-iT Cell Buffer Kit (Invitrogen) and 5 μm biotin azide (PEG4 carboxamide-6-azidohexanyl Biotin) (Invitrogen). The nucleus pellet was resuspended in 500 μl of click reaction and incubated for 1 h at 4 °C under constant rotation. After the click reaction, nuclei were pelleted at 800 RCF for 10 min at 4 °C and washed twice by gentle resuspension in PBS followed by centrifugation. PBS was removed, and the blue nucleus pellet was placed at −80 °C to freeze. The frozen pellet was thawed on ice and was resuspended in 550 μl of cold Buffer B1 (25 mm NaCl, 2 mm EDTA, 50 mm Tris-HCl, pH 8.0, and 1% IGEPAL CA630) and sonicated using a probe sonicator at 25 A for 20 min, 10 s on and 10 s off. Cell debris was pelleted by centrifuging at maximum speed for 10 min at 4 °C. 500 μl of supernatant was transferred to a new tube, and 500 μl of Buffer B2 (150 mm NaCl, 2 mm EDTA, 50 mm Tris-HCl, pH 8.0, and 0.5% IGEPAL CA630) was added to cleared supernatant. An aliquot (1:20) was saved for input. Dynabeads MyOne Streptavdin T1 (Thermo Fisher Scientific) were prepared by resuspending and washing beads with Buffer B2 by vortexing (50 μl/sample). A magnetic rack was applied to separate beads and remove wash buffer. Beads were resuspended in starting volume with Buffer B2. Beads were incubated with supernatant overnight at 4 °C. The next day, samples were washed three times with Buffer B2 and a final wash with Buffer B3 (150 mm NaCl, 2 mm EDTA, 50 mm Tris-HCl, pH 8.0). After removal of the last wash, the bead pellet and saved input were resuspended in DNA lysis buffer (2% SDS, 10 mm Tris, pH 7.4, and 10 mm EDTA). DNA was further purified by DNA chloroform extraction. The DNA pellet was resuspended in Tris-EDTA buffer, pH 8.0, and analyzed by qPCR.

### aniPOND

This method was adapted from previous aniPOND protocols (1, 72). Subconfluent HF, T-HF, T-HF, or HSV-TK-FLAG cells or HF cells infected with AD169 or HSV-KOS were pulsed and harvested as described for the FENDI protocol. Modifications include the addition of protease inhibitor mixture 1:100 (Millipore Sigma) to NEB, Click reaction, and Buffer B1. After sonication, lysate remained in Buffer B1. After incubation with T1 beads overnight, beads were washed with Buffer B1 2–4 times. After the final wash, beads were resuspended in Laemmli buffer with β-mercaptoethanol and boiled for 5 min before Western blot analysis as described previously. For HF cells infected with AD169 or HSV-KOS cells, the protocol was scaled up and performed with 60 × 10^6^ cells.

### qPCR

Nascent DNA from the subconfluent HF cells, HSV- or HCMV-infected HF cells, or subconfluent T-HF or T-HF HSV-TK-FLAG cells were isolated by following the FENDI protocol as described previously. The captured DNA was analyzed by qPCR using SsoAdvanced Universal Probes Supermix (Bio-Rad) along with Taqman primers and probes (Table S2). Samples were performed in triplicates in a standard 96-well plate. The following qPCR program was used: 1 cycle of 95 °C for 3 min and 40 cycles of 95 °C for 15 s and 60 °C for 30 s.

### Kinase assay

The phosphorylation activity of human thymidine kinase (R&D Systems, catalogue no. 8180-TK-050) and HSV thymidine kinase (MyBioSource, catalogue no. MBS1237171) on EdU, EdC, thymidine (Millipore Sigma), and 2-deoxycytidine (Millipore Sigma) was assayed according to the manufacturer's protocol using the Universal Kinase Activity Kit (R&D Systems, catalogue no. EA004). Each reaction in the coupled assay contained 4.5 ng/μl (0.225 μg) kinase, 2 ng/μl (0.1 μg) coupling phosphatase, 0.2 mm ATP, and 4.6 mm acceptor substrate. In the presence of zebularine (Millipore Sigma), coupling assay reactions contained 4.5 ng/μl (0.225 μg) kinase, 2 ng/μl (0.1 μg) coupling phosphatase, 0.2 mm ATP, 1.15 mm acceptor substrate, and 3.45 mm zebularine. Phosphate release was measured by determining optical density at 620 nm after a 20-min incubation. Specific activity was calculated with the following equation (49).

(Eq. 1) $$\text{Activity}\left(\text{pmol}/\min/\mu \text{g}\right)=\frac{\text{Adjusted phosphate released}\;\left(\text{nmol}\right)\times 1000\;\left(\text{pmol}/\text{nmol}\right)}{\text{Incubation time}\;\left(\min\right)\times \text{amount of enzyme}\;\left(\mu \text{g}\right)\times \text{coupling rate}}$$

For *K_m_* and *K*_cat_ calculations, experiments were performed to measure enzyme kinetics of HSV-TK and hTK in the presence of EdU and EdC using the ADP-Glo Kinase Assay Kit (Promega, catalogue no. V6930). Titration curves were performed for all substrates and kinases to determine optimal concentrations required for enzymatic assays. Luciferase activity from ADP production was measured using the Femtomaster FB15 (Zylux Inc.). To calculate the velocity of enzymatic activity, individual kinase reactions contained 4 ng/μl kinase, 30 μm ATP, and 0–225 μm EdU or EdC. Kinase reactions were performed at 37 °C for 0, 15, 30, 45, and 60 min. Luciferase activity resulting from ADP production was measured using the Hidex Chameleon microplate reader. Data from three technical replicates were plotted, and the generated curves were fitted to the Michaelis–Menten equation.

## Data availability

All of the data are reported and contained within the article and supporting material.

## Author contributions

S. M. and C. C. R. conceptualization; S. M. and C. C. R. formal analysis; S. M. and C. C. R. validation; S. M. and R. O. investigation; S. M. and C. C. R. visualization; S. M. and C. C. R. methodology; S. M. and C. C. R. writing-original draft; S. M. and C. C. R. writing-review and editing; C. C. R. resources; C. C. R. supervision; C. C. R. funding acquisition; C. C. R. project administration.

## Supplementary Material

Supporting Information

## Footnotes

3 J. Dembowski, personal communication.

## Footnotes

2 The abbreviations used are:

BrdU: 5-bromo-2′-deoxyuridine
EdU: 5-ethynyl-2′-deoxyuridine
EdC: 5-ethynyl-2′-deoxycytidine
UNG: uracil-DNA glycosylase
vUNG: viral UNG
iPOND: isolation of proteins on nascent DNA
aniPOND: accelerated native iPOND
HSV: herpes simplex virus
HF: human fibroblast
HCMV: human cytomegalovirus
KSHV: Kaposi's sarcoma-associated herpesvirus
hpi: hours postinfection
FENDI: fast and efficient nascent DNA isolation
qPCR: quantitative PCR
MTT: 3-(4,5-dimethylthiazol-2-yl)-2,5-diphenyltetrazolium bromide
RPE cell: retinal pigmented epithelial cell
GCV: ganciclovir
ACV: acyclovir
T-HF: life-expanded telomerized human fibroblast
TK: thymidine kinase
hTK: human TK
MOI: multiplicity of infection
DAPI: 4′,6-diamidino-2-phenylindole
TPA: 12-*O*-tetradecanoylphorbol-13-acetate
NEB: nucleus extraction buffer
RCF: relative centrifugal force
ANOVA: analysis of variance.
