# Supplementary material for: 5-Ethynyl-2′-deoxycytidine and 5-ethynyl-2′-deoxyuridine are differentially incorporated in cells infected with HSV-1, HCMV, and KSHV viruses
Source: J Biol Chem. 2020 Mar 23;295(18):5871–90. doi: 10.1074/jbc.RA119.012378 (PMC7196651; doi:10.1074/jbc.RA119.012378)
Supplement: Supporting Information [file supp_295_18_5871__index.html]

5-Ethynyl-2’-deoxycytidine and 5-ethynyl-2’-deoxyuridine are differentially incorporated in cells infected with HSV-1, HCMV, and KSHV viruses — Differential incorporation of EdU and EdC into nascent DNA — 5-Ethynyl-2′-deoxycytidine and 5-ethynyl-2′-deoxyuridine are differentially incorporated in cells infected with HSV-1, HCMV, and KSHV viruses — Differential incorporation of EdU and EdC into nascent DNA — Supporting Information 

# 5-Ethynyl-2′-deoxycytidine and 5-ethynyl-2′-deoxyuridine are differentially incorporated in cells infected with HSV-1, HCMV, and KSHV viruses

## Supporting Information

- Supporting Information (to be published online) - Supporting figures and tables
