# Supplementary material for: 5-Ethynyl-2′-deoxycytidine and 5-ethynyl-2′-deoxyuridine are differentially incorporated in cells infected with HSV-1, HCMV, and KSHV viruses
Source: J Biol Chem. 2020 Mar 23;295(18):5871–90. doi: 10.1074/jbc.RA119.012378 (PMC7196651; doi:10.1074/jbc.RA119.012378)
Supplement: Supporting Information [file supp_RA119.012378_157819_2_supp_495890_q7nvmc.pdf]

Supporting Information Figure S1. Super-confluent HF cells do not incorporate EdU. HF cells were pulse labeled with EdU for 30 minutes. Cells were fixed and labeled nascent DNA was tagged with Alexa Fluor 594 (red). Nuclei were stained with DAPI (blue). Imaging was performed at 10x magnification. (A) HFs were grown to super confluency to inhibit DNA replication. Cells were pulsed at four, five, six, seven, or eight days post plating. (B) Sub-confluent HF cells were pulsed from 18-24 hours post-plating.

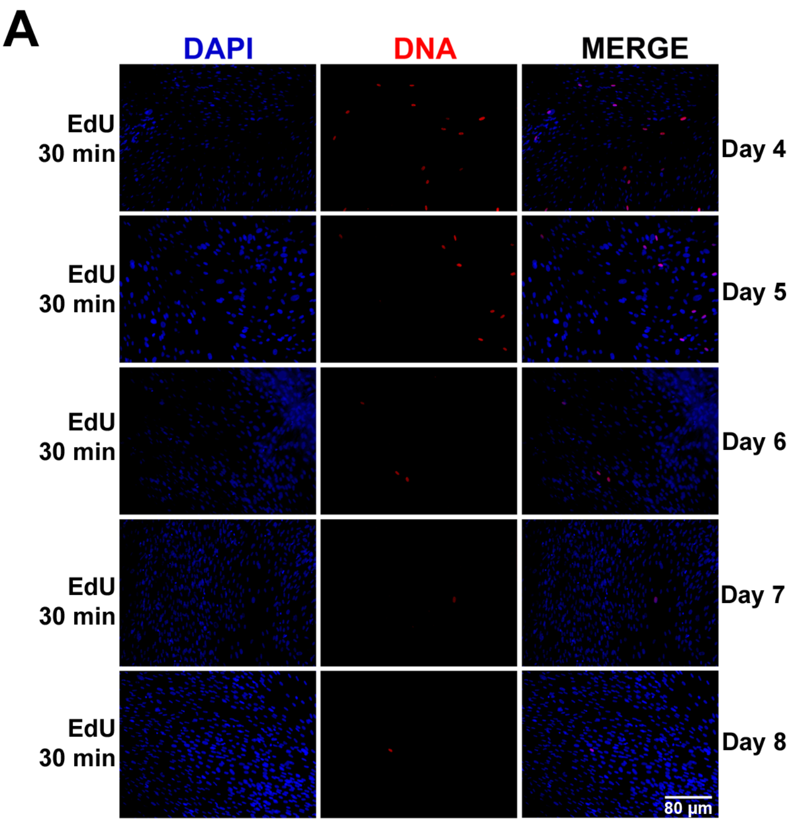

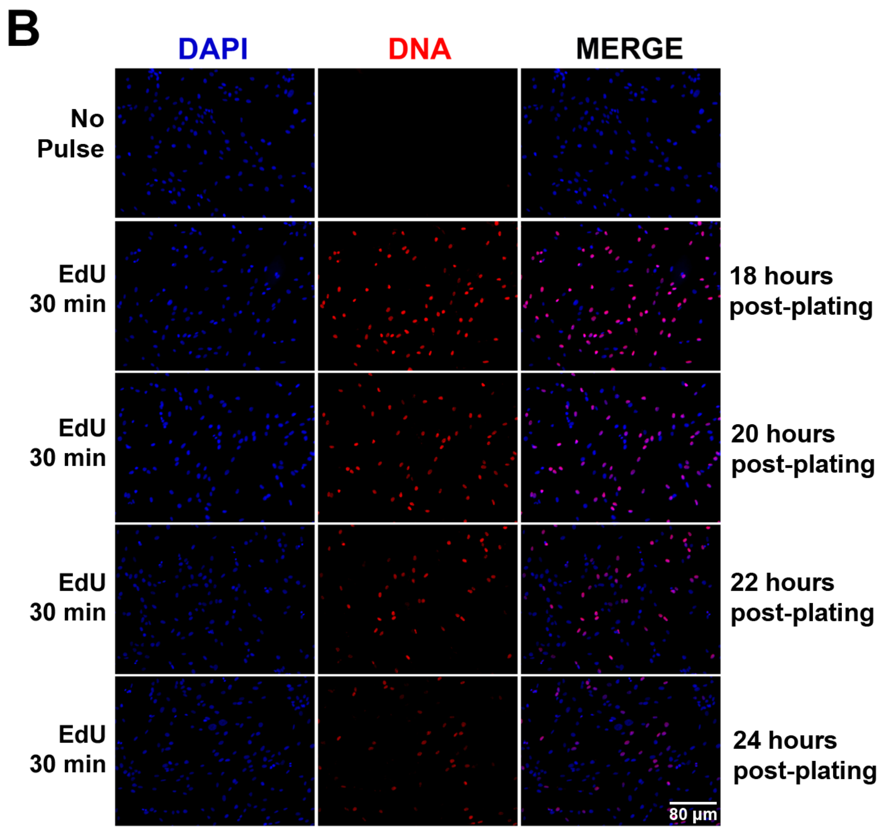

Supporting Information Figure S2. HF cells pulsed for 30 minutes with EdU or EdC at 100% confluency versus 50% confluency. Super-confluent (100%) and sub-confluent (50%) HFs were pulse labeled with EdU or EdC for 30 minutes. Cells were fixed and labeled nascent DNA was tagged with Alexa Fluor 594 (red). Nuclei were stained with DAPI (blue). Cells were imaged at 40x (upper panel) and 10x (lower panel) magnification.

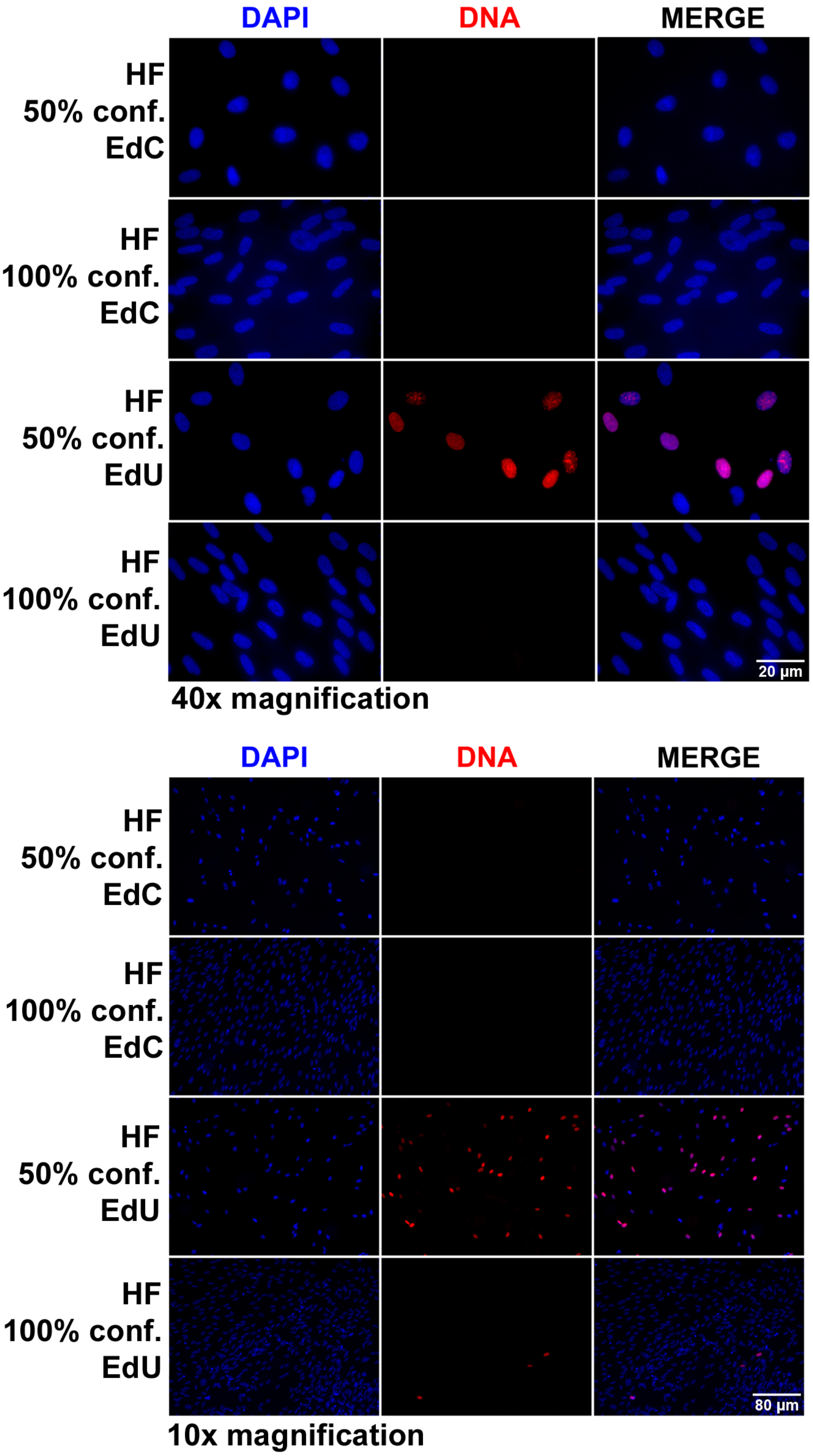

Supporting Information Table S1. Sequences for PCR primers and gBlocks

Gene		Sequence 5’- 3’
HSV-TK FLAG	gBlock:	CGTGAGGATCTATTTCCGGTGAATTCGCCACCATGGCTTCGTACCCCTGCCATCAA<br>CACGCGTCTGCGTTCGACCAGGCTGCGCGTTCTCGCGGCCATAGCAACCGACGT<br>ACGGCGTTGCGCCCTCGCCGGCAGCAAGAAGCCACGGAAGTCCGCCTGGAGCAG<br>AAAATGCCCACGCTACTGCGGGTTTATATAGACGGTCCTCACGGGATGGGGAAAA<br>CCACCACCACGCAACTGCTGGTGGCCCTGGGTTCGCGCGACGATATCGTCTACGT<br>ACCCGAGCCGATGACTTACTGGCAGGTGCTGGGGGCTTCCGAGACAATCGCGAA<br>CATCTACACCACACAACACCGCCTCGACCAGGGTGAGATATCGGCCGGGGACGC<br>GGCGGTGGTAATGACAAGCGCCCAGATAACAATGGGCATGCCTTATGCCGTGACC<br>GACGCCGTTCTGGCTCCTCATATCGGGGGGGAGGCTGGGAGCTCACATGCCCCG<br>CCCCCGGCCCTCACCCTCATCTTCGACCGCCATCCCATCGCCGCCCTCCTGTGCT<br>ACCCGGCCGCGCGATACCTTATGGGCAGCATGACCCCCCAGGCCGTGCTGGCGT<br>TCGTGGCCCTCATCCCGCCGACCTTGCCCGGCACAAACATCGTGTTGGGGGCCCT<br>TCCGGAGGACAGACACATCGACCGCCTGGCCAAACGCCAGCGCCCCGGCGAGCG<br>GCTTGACCTGGCTATGTTGGCCGCGATTCGCCGCGTTTACGGGCTGCTTGCCAAT<br>ACGGTGCGGTATCTGCAGGGCGGCGGGTCGTGGCGGGAGGATTGGGGACAGCT<br>TTCGGGGACGGCCGTGCCGCCCCAGGGTGCCGAGCCCCAGAGCAACGCGGGCC<br>CACGACCCCATATCGGGGACACGTTATTTACCCTGTTTCGGGCCCCCGAGTTGCT<br>GGCCCCCAACGGCGACCTGTATAACGTGTTTGCCTGGGCCTTGGACGTCTTGGCC<br>AAACGCCTCCGTCCCATGCACGTCTTTATCCTGGATTACGACCAATCGCCCGCCG<br>GCTGCCGGGACGCCCTGCTGCAACTTACCTCCGGGATGGTCCAGACCCACGTCA<br>CCACCCCAGGCTCCATACCGACGATCTGCGACCTGGCGCGCACGTTTGCCCGGG<br>AGATGGGGGAGGCTAACGATTACAAGGATGACGACGATAAGTGATCTAGAGCGGC<br>CGCGGATCCCGCCCC
intron-MCS-TKpolyA	gBlock:	TTCCATTTCAGGTGTCGTGAAGGTAGCCTTGCAGAAGTTGGTCGTGAGGCACTGG<br>GCAGGTAAGTATCAAGGTTACAAGACAGGTTTAAGGAGACCAATAGAAACTGGGCT<br>TGTCGAGACAGAGAAGACTCTTGCGTTTCTGATAGGCACCTATTGGTCTTACTGAC<br>ATCCACTTTGCCTTTCTCTCCACAGGTGTCCACTCCCAGTTCAATTACAGCTCTTAA<br>AAATTGGATCTCCATTCGCCATTCAGGCTGCGCAACTGCTGGGAAGGACGATCAG<br>AGCGGGCCTCTTCGCTATTACGCCAGCTGGCGAAAGGGACGTGGCAAGCAAGGC<br>GATTAAGTTGAGTTACGCCAGGATTTTCCCAGTCACGACGTTGTAAAACGACGGCC<br>AGAGAATTATAATACGACTCACTATAGGGCGAATTGGGCCCGACGTCGCATGCTC<br>CTCTAGACTCGAGGAATTCGGCTTGGATCCGGAGAGCTCCCAACGCGTTGGATGC<br>ATCAAGCTTGAGACGGCAATAAAAAGACAGAATAAAACGCATTGAAGACGAAAGGG<br>CCTCG
EF1α	Forward:	CAATGTATCTTATCATGTCTGAGTAATTCATACAAAAGGA
	Reverse:	CAACTTCTGCAAGGCTACCTTCACGACACCTGAAATGGAA
HSV-TK	Forward:	CATGCTCCTCTAGACTCGAGGAATTCGCCACCATGGCTTCGTACCCCTGCCA
	Reverse:	TCCTCGCCCTTGCTCACCATGTTAGCCTCCCCCATCTCCC
GFP	Forward:	GGGAGATGGGGGAGGCTAACATGGTGAGCAAGGGCGAGGA
	Reverse:	AACGCGTTGGGAGCTCTCCGGATCCTCATTACTTGTACAGCTCGT

Supporting Information Table S2. Sequences for qPCR primers and probes

Gene		Sequence 5'- 3'
7SK	Forward:	TGACTACCCTACGTTCTCCTAC
	Reverse:	GTCAAGGGTATACGAGTAGCTG
	Probe:	56-FAM/CCCTGCTAG/ZEN/AACCTCCAAACAAGCT/3IABkFQ/
UL86	Forward:	TGCGTAAAGTCGAAGAAGGG
	Reverse:	CGCACGGTGAACGAAATAAAG
	Probe:	56-FAM/CAAGGTGGG/ZEN/CAACATCACGCTCTA/3IABkFQ/
UL19	Forward:	CTGGGTGAGCGTGAAGTTTA
	Reverse:	GACCGCTTTGTGACTGAGAA
	Probe:	/56-FAM/TGGAGCTGG/ZEN/CCCAGGAAGTA/3IABkFQ/
